# Acetatobis(l-arginine)copper(ii) acetate trihydrate: synthesis from l-argininium acetate, structural-electronic insights and *in vitro* antimicrobial activity

**DOI:** 10.1039/d6ra03860c

**Published:** 2026-08-27

**Authors:** Amani Direm, Salima Samai, Hamza Athmani, Mohammed S. M. Abdelbaky, Cemal Parlak, Olufunso Abosede, Santiago Garcia-Granda, Ponnadurai Ramasami

**Affiliations:** a Laboratory of Structures, Properties and Interatomic Interactions LASPI^2^A, Faculty of Sciences and Technology, Abbes Laghrour University Khenchela 40000 Algeria direm.amani@univ-khenchela.dz amani_direm@yahoo.fr +213 772 33 02 87; b Department of Matter Sciences, Faculty of Sciences and Technology, Abbes Laghrour University Khenchela 40000 Algeria; c Departamento de Química Física, Facultad de Ciencias Químicas, Universidad de Salamanca E-37008 Salamanca Spain; d Departamento de Química Física y Analítica, Universidad de Oviedo-CINN 33006 Oviedo Spain; e Department of Physics, Science Faculty, Ege University Izmir 35040 Turkey; f Department of Chemistry, Federal University Otuoke P.M.B 126 Yenagoa Bayelsa State Nigeria; g Computational Chemistry Group, Department of Chemistry, Faculty of Science, University of Mauritius Réduit 80837 Mauritius p.ramasami@uom.ac.mu +230 4037507; h Centre for Natural Product Research, Department of Chemical Sciences, University of Johannesburg Doornfontein 2028 South Africa

## Abstract

Acetatobis(l-arginine)copper(ii) acetate trihydrate, [Cu(OAc)(Arg)_2_](OAc)·3H_2_O (compound II), was synthesized *via* a complexation reaction using l-argininium acetate (compound I) as the precursor. Single-crystal X-ray diffraction analysis showed that both compounds crystallize in the monoclinic space group *P*2_1_. A detailed structural analysis revealed a robust 3D hydrogen-bonding network, where lattice water molecules and free acetate anions act as structural bridges, stabilizing the guanidinium side chains of the arginine ligands. A thorough Hirshfeld surface (HS) analysis, quantifying the contribution of N–H⋯O and O–H⋯O H-bonds in addition to non-classical H⋯H, H⋯C/C⋯H and H⋯N/N⋯H contacts to the overall lattice stability, was employed to explore the intermolecular interactions within the crystal structure in comparison with DUYCAB's. The electronic properties and the nature of the bonds in (II) were investigated using topological analysis (QTAIM). Furthermore, reactivity descriptors, molecular electrostatic potential (MEP), electron localization function (ELF), localized orbital locator (LOL), non-covalent interaction index (NCI) and reduced density gradient (RDG) of the studied complex were calculated and analyzed. Finally, the biological behaviors of complex (II) and its starting material (I) were evaluated by carrying out *in vitro* antimicrobial assays against a panel of pathogenic bacterial and fungal strains. The results demonstrate significant inhibitory activity, suggesting that the integration of the bioactive l-arginine moiety with the copper(ii) centers may lead to enhancing the pharmacological profile of the precursor. This work provides a structural and theoretical framework for the development of arginine-based metal complexes as candidates for next-generation antimicrobial drugs.

## Abstract 2

Acetatobis(l-arginine)copper(ii) acetate trihydrate shows strong coordination, H-bonding and non-classical interactions *via* SCXR diffraction, HS, ELF/LOL and QTAIM analyses. It also displays significant *in vitro* antimicrobial activity.

## Graphical abstract

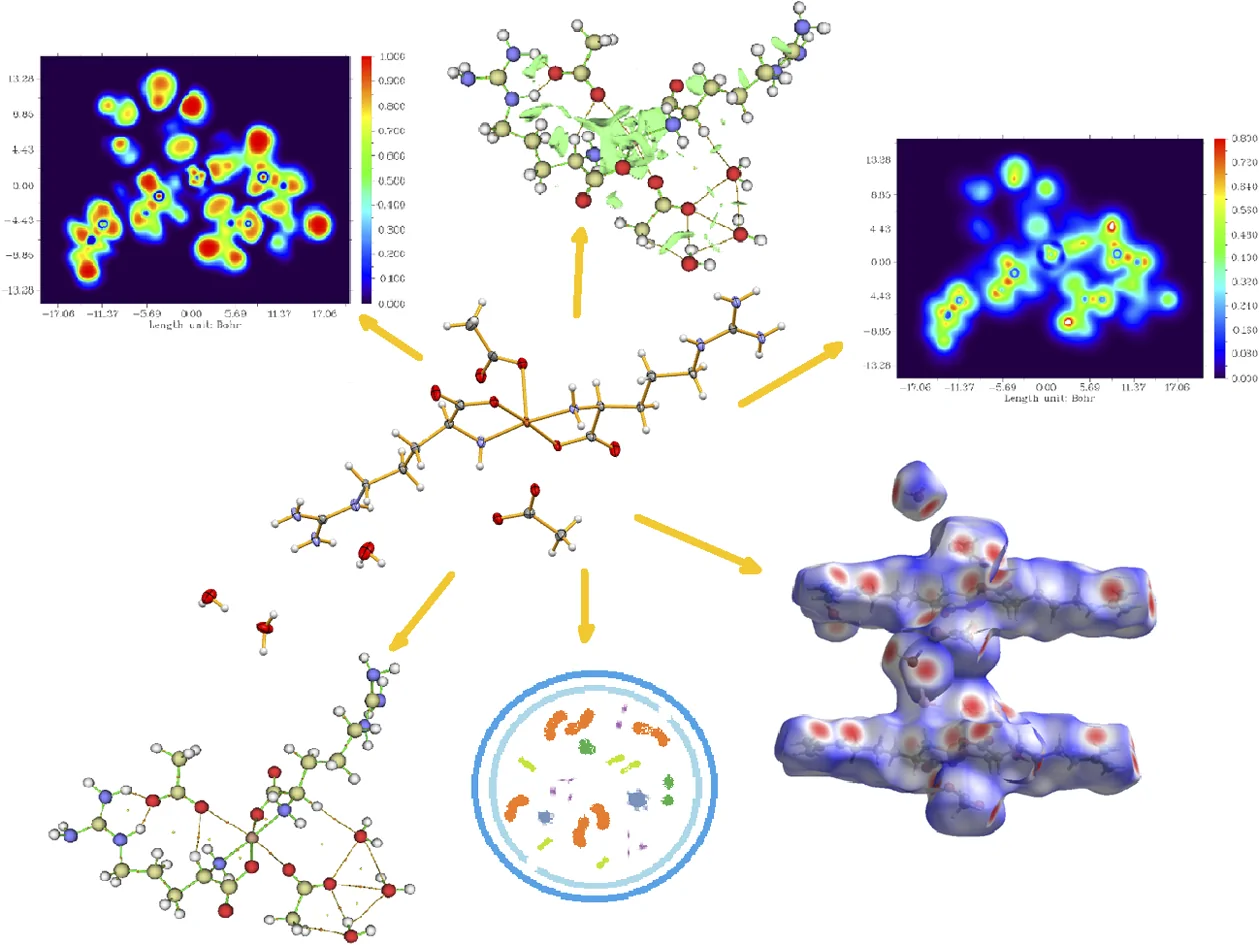

## 1. Introduction

The design and synthesis of new amino acid-based metal coordination complexes represent a major frontier in inorganic and bioinorganic chemistry, lying at the intersection of the biological sciences and advanced materials science. Among amino acids (AAs), 2-amino-5-guanidinopentanoic acid, commonly known as l-arginine, is one of the basic AAs encoded by DNA. Owing to its favorable physicochemical properties, l-arginine has also been employed as a nonlinear optical (NLO) material in optoelectronic applications.^1–3^ Furthermore, the presence of the guanidine group in l-arginine is crucial in the context of structural similarity to two antibiotics – netropsin and distamycin^4,5^, which also can act as effective photo-synthesizers.^6^

On the other hand, copper(ii) coordination complexes with biologically relevant ligands have long been studied in coordination chemistry due to their diverse geometries. Furthermore, copper is an essential trace element that participates in electron transfer, oxidative stress processes, and enzymatic catalysis in biological systems, and its complexes have demonstrated promising antimicrobial, DNA-binding and therapeutic properties.^7^ In this context, l-arginine is a good chelating agent and exhibits strong coordination ability with metal ions. In particular, its copper(ii) complexes display diverse structural topologies and supramolecular self-assembly, attracting considerable attention because of their cooperative magnetic behavior and intriguing spectroscopic properties.^8–10^

Among the fifty l-arginine metal complexes reported in the Cambridge Structural Database (CSD, ConQuest version 2023.10), thirty-nine contain copper centers, highlighting the pronounced affinity of this AA for Cu(ii) ions. Furthermore, five of these structures exhibit close structural similarity to the complex reported herein and crystallize in the same monoclinic space group, *P*2_1_. These include the structures with refcodes DAFYIW,^11^KIXPIR,^12^EBAWAH,^13^LOHWUD (ref. 14) and DUYCAB.^15^ Two of the mentioned complexes contain Zn(ii) centers coordinated to two l-arginine ligands, whereas the remaining three comprise Cu(ii) centers. Moreover, the copper(ii) complexes differ in their counter-ions, namely nitrate in DAFYIW and KIXPIR, acetate in EBAWAH, and isothiocyanate in LOHWUD. Among them, DUYCAB exhibits the biggest structural similarity to the title complex with respect to both the coordination environment and the ligand set. However, it differs in the relative positioning of molecules within the asymmetric unit, leading to a distinct packing arrangement. This highlights the structural versatility of l-arginine in metal coordination chemistry, while underlining the influence of the auxiliary ligand and asymmetric unit organization on the resulting crystal architecture.

In this context and as part of our studies on heterocyclic-based systems with promising biological properties,^16–19^ we describe herein the preparation from l-argininium acetate (compound (I) hereafter), the crystal structure description, Hirshfeld surface analysis, quantum theory of atoms in molecules (QTAIM) analysis and *in vitro* antimicrobial assays of acetatobis(l-arginine)copper(ii) acetate trihydrate, abbreviated as (II) hereafter.

## 2. Materials and methods

### 2.1. Synthesis

#### 2.1.1. l-Arginiunium acetate (I)

l-Argininium acetate Arg.OAc, or compound (I) hereafter, was prepared by reacting l-arginine (0.174 g, 1 mmol), previously dissolved in a 10 ml combination of water/methanol (1 : 1), with 5 ml acetic acid. The mixture was then heated at 60 °C for 30 minutes and left to slowly evaporate at room temperature. Three days later, colorless single crystals were obtained.

#### 2.1.2. Acetatobis(l-arginine) copper(ii) acetate trihydrate (II)

Ligand (I) (0.468 g, 2 mmol) was dissolved in a 15 ml water/methanol solution (2 : 1) and a solution of CuSO_4_·5H_2_O (0.250 g, 1 mmol) in 10 ml water/methanol (1 : 1) was added drop-wise. The resulting mixture was then heated under reflux for three hours and filtered. Light-blue block-like single-crystals of acetatobis(l-arginine)copper(ii) acetate trihydrate [Cu(OAc)(Arg)_2_](OAc)·3H_2_O, abbreviated as compound (II) hereafter, were obtained after two weeks of slow evaporate at room temperature.

### 2.2. Single-crystal X-ray diffraction

The single-crystal X-ray diffraction data of (I) and (II) were collected at room temperature (293 K) on an Rigaku Xcalibur Gemini diffractometer, equipped with a monochromatic Mo Kα radiation (*λ* = 0.71073 Å) tube. The structures of (I) and (II) were solved using the direct methods implemented in the program Sir2019 (ref. 20) and refined by a full-matrix least-squares’ technique based on *F*^2^ using the program ShelXL-2014.^21^ All the H atoms were located in the difference Fourier maps and treated by a mixture of independent/constrained refinement. The crystals' details, data collection and refinement parameters for (I) and (II) are listed in Table 1. The molecular structure plots were prepared using Mercury software (Version 2023.1.0).^22^ The X-ray coordinates and basic geometrical data were deposited at the Cambridge Crystallographic Data Centre (CCDC), with CCDC deposition numbers 2523709 (I) and 2523710 (II). The data can be obtained free of charge from The Cambridge Crystallographic Data Centre *via*https://www.ccdc.cam.ac.uk/data_request/cif.

**Table 1:** Crystal information, data collection and structure refinement of (I) and (II)

Crystal data	(I)	(II)
Chemical formula	C_8_H_18_N_4_O_4_	C_16_H_40_CuN_8_O_11_
Formula weight (g mol^−1^)	234.26	584.10
Crystal system, space group	Monoclinic, *P*2_1_	Monoclinic, *P*2_1_
Temperature (K)	293	293
*a*, *b*, *c* (Å)	9.231(2), 5.170(2), 13.100(3)	10.3040(2), 16.7756(3), 15.7774(4)
*α*, *β*, *γ* (°)	90, 109.90(1), 90	90, 107.455(2), 90
Volume (Å^3^)	588(3)	2601.63(10)
*Z*	2	4
Absorption coefficient (mm^−1^)	0.11	0.91
Crystal size (mm^3^)	0.10 × 0.11 × 0.13	0.10 × 0.11 × 0.15
Color	Colorless	Light-blue
Shape	Prism	Block-like crystal

**Data collection**		
Diffractometer	Rigaku Xcalibur Gemini	Rigaku Xcalibur Gemini
Radiation type	Mo Kα	Mo Kα
No. of measured, independent and observed [*I* > 2*σ*(*I*)] reflections	11 381, 2706, 2332	38 398, 17 269, 11 868
*R*_int_	0.049	0.049
*θ*_max_, *θ*_min_ (°)	27.5, 3.3	31.5, 3.2
*h*, *k*, *l*	−11 → 10, −6 → 6, −17 → 17	−15 → 14, −22 → 24, −23 → 23

**Refinement**		
*R*[*F*^2^ > 2*σ*(*F*^2^)], w*R*(*F*^2^), *S*	0.057, 0.164, 1.13	0.045, 0.091, 1.0
No. of reflections	2706	17 269
No. of parameters	146	653
H atoms' treatment	H atoms treated by a mixture of independent and constrained refinement	H atoms treated by a mixture of independent and constrained refinement
Δ*ρ*_max_, Δ*ρ*_min_ (e Å^−3^)	0.30, −0.38	0.47, −0.55

### 2.3. Computational details

#### 2.3.1. Hirshfeld surface analysis

The concept of Hirshfeld surface (HS) analysis and its associated 2D-fingerprint plots (FPs) are of growing significance for a complete understanding of structural descriptions. The purpose of building HSs is an attempt to specify the space occupied by a molecule in a crystal in order to divide the electron density of the crystal into small fragments of electron density. In the HS analysis, each point on the isosurface is defined by two distances, *d*_e_ and *d*_i_, corresponding to the distances to the nearest nuclei external and internal to the surface, respectively. Their mathematical combination enables color-coded visualization of intermolecular contact features. In this concept, the color scale reflects the presence of possible hydrogen bonds and van der Waals interactions. Spots on the HS correspond to distances between two considered atoms: bright red denotes shorter than, white equal to, and blue longer than the sum of their van der Waals (vdW) radii. Furthermore, 2D-FPs allow summarizing the contact distances to the HS (*d*_e_, *d*_i_) and demonstrate to be characteristic of particular close-contact environments. The HS and 2D-FP analyses presented in this study were carried out using CrystalExplorer (version 3.1)^23^ on the crystal structures of compounds (I) and (II).

#### 2.3.2. DFT calculations and AIM analyses

A full geometry optimization was performed in the gas phase for complex (II) using density functional theory (DFT) as implemented in the Gaussian 09 software package.^24^ The calculations were carried out for the d^9^ Cu(ii) complex in its +2 charge state. Owing to the open-shell nature of the system, all calculations were performed using the unrestricted B3LYP formalism. The spin state was explicitly defined as a doublet (spin multiplicity = 2), and the expectation value of 〈*S*^2^〉 before spin annihilation (≈0.75) confirmed negligible spin contamination.

The geometry considered for the calculation was obtained from the experimental single-crystal structure. All calculations were performed at the generalized gradient approximation (GGA) level, with the B3LYP^25^ exchange-correlation functional with the LanL2DZ basis set.^26^ The vibrational frequency calculations were performed to verify that the optimized geometry represents local minima. The molecular systems studied herein were first structured using the Avogadro software.^27^

On the other hand, the QTAIM method^28^ is an effective theory for analyzing and characterizing chemical bonds. The properties associated with the critical points (CPs) (3, −1) and the electron density characterizations describe the chemical bond, which made it possible to determine the nature of the bonds in (II). The QTAIM analysis was performed to search for all the bonds' critical points (BCPs) using the Multiwfn software.^29^ The Non-Covalent Interaction index (NCI) and Reduced Density Gradient (RDG) analyses (NCI-RDG) were performed using the VMD software.^30^

The reactivity descriptors, including the gap energy, ionization potential (*I*), electron affinity (*A*), chemical potential (*µ*), hardness (*η*), flexibility (*S*), electronegativity (*χ*) and global electrophilicity index (*ω*), were all examined according to Koopmans' theorem.^31^ The descriptors were computed based on the equations reported in the literature.^32–35^

### 2.4. Antimicrobial assays

#### 2.4.1. Microbial isolates

Two clinical bacterial isolates (*Staphylococcus aureus* and *Escherichia coli*) and two clinical fungal isolates (*Candida albicans* and *Aspergillus niger*) were obtained from the laboratory unit St. Luke's Hospital Anua in Uyo, AkwaIbom State (Nigeria). The isolates were purified by sub-culturing and biochemical tests were performed to confirm their identity prior to use in the assays.

#### 2.4.2. Preparation of the test isolates

The pure cultures of the test organisms were inoculated into a broth medium and incubated at 37 °C for 24 hours for the bacterial isolates and 72 hours for the fungal isolates. In order to standardize the inoculum size, the cultures were subjected to 10-fold serial dilutions. *S. aureus*, *C*. *albican* and *A. niger* were diluted to a factor of 10^−3^, whereas *E. coli* was diluted to a factor of 10^−5^, following the established protocols by Ekong *et al.*^36^

#### 2.4.3. Screening of the studied compounds for antimicrobial activity

Compounds (I) and (II) were screened for antimicrobial activity using the agar well-diffusion technique, as described by Okeke *et al.*^37^ Briefly, 0.5 ml of each of the test organisms was aseptically spread onto the surface of Müller–Hinton agar (MHA) plates using a sterile hockey stick. The plates were allowed to stand for 30 minutes, in order to permit pre-diffusion of the inoculum into the medium. A sterile 5 mm cork borer was then used to bore holes into the agar.

Then, the studied compounds were dissolved in dimethyl sulfoxide (DMSO) and subjected to a two-fold serial dilution to reach a dilution factor of 2^−3^ (up to the third dilution). All the antimicrobial tests were performed at four different concentrations (20, 10, 5 and 2.5 mg mL^−1^) across independent biological runs, with the final consistent datasets evaluated in duplicate.

A 0.5 ml volume of each diluted compound was introduced into the wells. The plates were left to stand undisturbed at room temperature for 1 hour, in order to allow the compounds to diffuse into the agar before the incubation. Chloramphenicol (Chl) was used as a standard reference drug for the antibacterial activity, while Nystatin (Nyst) served as the standard for the antifungal activities. All plates were incubated at 37 °C for 24 (bacteria) and 72 hours (fungi). The antimicrobial activity was determined by measuring the diameters of the inhibition zones in millimeters (mm) for each concentration.

#### 2.4.4. Determination of minimum inhibitory concentration (MIC)

The Minimum Inhibitory Concentration (MIC) was determined for the compounds that exhibited the smallest zones of inhibition during the initial screening. These compounds were subjected to a two-fold serial dilution in broth and each tube was then inoculated with 0.1 mL from the standardized microbial suspension. A control experiment was simultaneously prepared, consisting of the growth medium and inoculum, without adding the studied compounds. All tubes were incubated at 37 °C for 24 hours (for bacteria) and 72 hours (for fungi). The MIC values were then determined visually, by considering the lowest concentration of the compound that showed no visible turbidity, which indicates the complete inhibition of microbial growth.

## 3. Results and discussion

### 3.1. Crystal structure

The single-crystal X-ray diffraction analysis showed that both the ligand (I) and its complex (II) crystallize in the monoclinic space group *P*2_1_. The asymmetric unit (a.u.) of (I) comprises an argininium cation and an acetate anion. Whereas the a.u. of (II) consists of two crystallographically-independent units; each containing a mononuclear [Cu(OAc)(Arg)_2_]^+^ cation, an uncoordinated acetate counter-ion and three water molecules. In the [Cu(OAc)(Arg)_2_]^+^ cations, each of the copper(ii) centers (Cu1 and Cu2) is coordinated to N and O donor atoms from two bidentate arginine ligands, in addition to an oxygen atom from the acetate anion. Both Cu(ii) centers adopt a distorted square-pyramidal geometry, where the basal plane is defined by two *trans-N*,*O* chelated l-arginine molecules. The related Cu–O and Cu–N bond lengths fall within the typical range observed for Cu(ii) complexes with mixed O/N coordination environments'. Their values vary from 1.948(2) to 1.958(2) Å and from 1.985(2) to 2.000(2) Å, respectively. Similarly, the related equatorial bond lengths reported for DUYCAB^15^ range from 1.936(12) to 1.944(13) Å and from 1.986(15) to 1.996(13) Å, respectively.

Furthermore, the apical position is occupied by an oxygen atom from an acetato anion (O5 for Cu1 and O16 for Cu2), with CuO bonds of 2.512(2) and 2.469(2) Å. However, the non-coordinated Cu⋯O acetate counter-ion distances (O7 for Cu1 and O13 for Cu2) were found to be 2.871(2) Å and 2.848(2) Å, respectively. Therefore, it is worth noting that the environment around Cu2 is slightly more compressed compared to Cu1. Also, the axial CuO bond lengths in DUYCAB are 2.570(15) Å (molecule A) and 2.563(16) Å (molecule B), whereas the non-coordinated Cu⋯O lengths are 2.884(14) Å for molecule A and 2.944(16) Å for molecule B.

**Fig. 1:**
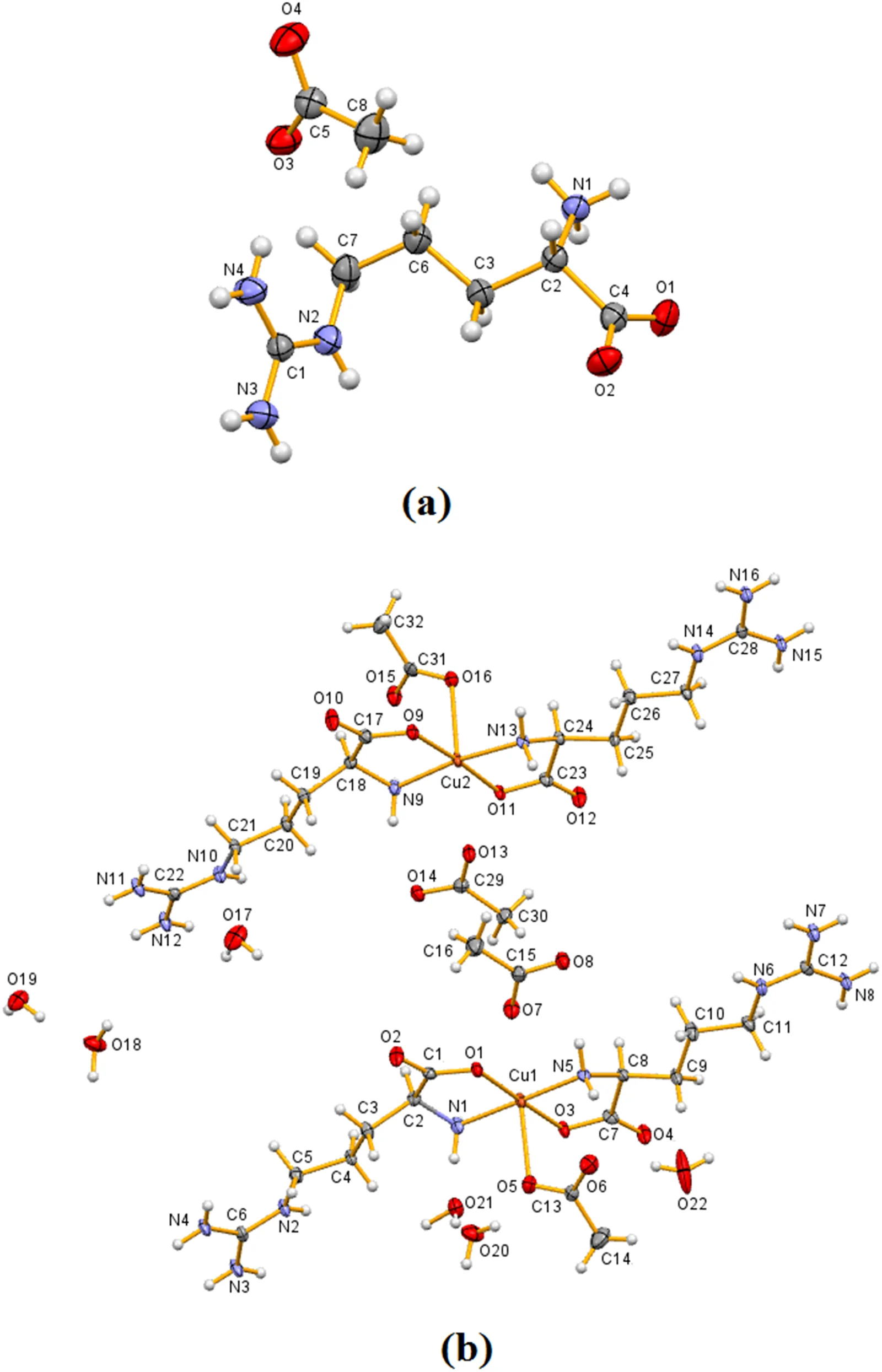
Asymmetric units of (a) the ligand (I) and (b) its complex (II).

The molecular structures and atom numbering of (I) and (II) are shown in Fig. 1. The hydrogens' numbering was omitted for clarity and the thermal ellipsoids were drawn at the 30% probability level.

**Fig. 2:**
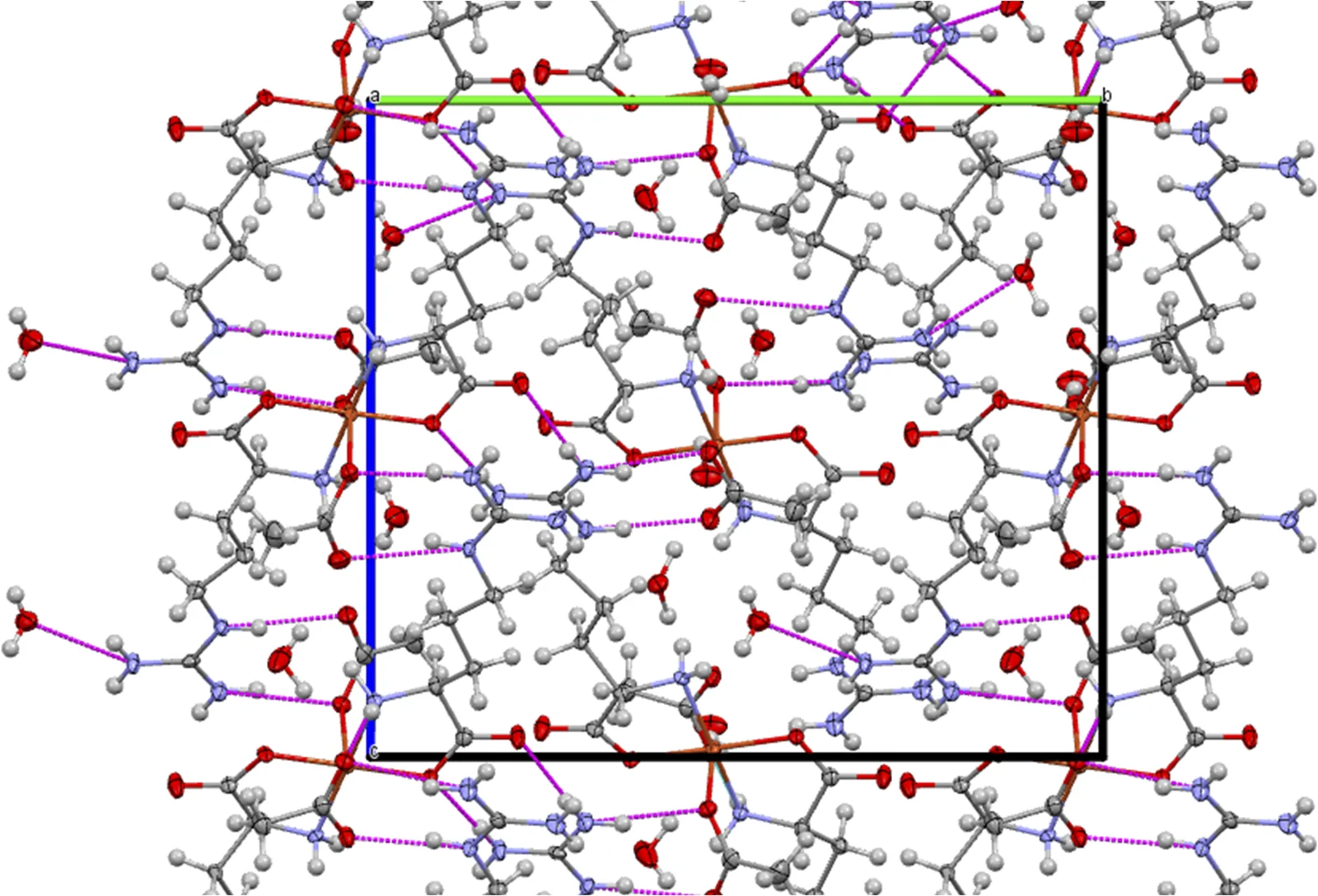
Projection of the crystal packing through the (*bc*) plane showing the H-bonds in (II).

The stability of complex (II) is governed by a dense network of N–H⋯O and O_W_–H_W_···O hydrogen bonds (see Table 2 and Fig. 2). Indeed, the guanidinium moieties act as glue by forming multiple H-bonds with the oxygens of the non-coordinated acetate groups, namely N3–H3E⋯O14; 2.980(3) Å, N16–H16D⋯O8; 2.904(3) Å, N14–H14D⋯O7; 2.863(3) Å and N2–H2E⋯O13; 2.844(3) Å. Furthermore, the coordinated acetato groups bridge the copper units together by bonding to the guanidinium moieties through N–H⋯O H-bonds (for exp. N12–H12E⋯O5; 2.851(4) Å). The ligands' carboxylate groups also help stabilizing the coordination sphere *via* shorter H-bonds linking the guanidinium side chains (N3–H3D⋯O12; 2.796(3) Å, N12–H12D⋯O4; 2.840(3) Å, N16–H16E⋯O2; 2.791(3) Å and N7–H7E⋯O10; 2.805(3) Å). It is worth noting that the water molecules act as a bridge linking the coordinated and non-coordinated acetate groups to each other through O_W_–H_W_⋯O hydrogen bonds, for exp. (O20–H8W⋯O8; 2.721(2) Å with O20–H7W⋯O5; 2.673(2) Å), (O22–H11W⋯O14; 2.819(3) Å with O22–H12W⋯O6; 2.726(3) Å) and (O18–H3W⋯O16; 2.666(3) Å with O18–H4W⋯O14; 2.771(2) Å).

**Table 2:** Geometric parameters of the H-bonds in (I) and (II)^a^

D–H⋯A	D–H (Å)	H⋯A (Å)	D⋯A (Å)	D–H⋯A (°)
**(I)**				
N4–H4A⋯O3	0.86	2.01	2.819(8)	156
N1–H1A⋯O1	0.89	1.97	2.615(3)	167
N1–H1A⋯N3^i^	0.89	2.63	3.328(4)	136
N2–H2⋯O2^ii^	0.86	2.00	2.834(3)	164
N2–H2⋯O1^ii^	0.86	2.90	3.350(4)	114
N3–H3BN···O1^iii^	0.86	2.36	2.919(4)	123
N1–H1C⋯O4^iv^	0.89	1.91	2.701(4)	146
N3–H3AN⋯O4^v^	0.86	1.96	2.821(4)	176

**(II)**				
N9–H9D⋯O15	0.90	2.58	3.150(3)	122
N9–H9E⋯O13	0.90	2.94	3.388(3)	113
N9–H9E⋯O14	0.90	2.12	2.971(3)	158
N13–H13E⋯O13	0.90	2.90	3.351(3)	113
N13–H13E⋯O21	0.90	2.24	3.106(3)	161
N1–H1D⋯O5	0.90	2.76	3.139(3)	107
N1–H1E⋯O21^i^	0.90	2.83	3.339(3)	117
N1–H1D⋯O20^i^	0.90	2.05	2.904(3)	158
N1–H1D⋯O21^i^	0.90	2.98	3.339(3)	106
N5–H5D⋯O8	0.90	2.64	3.275(3)	128
N5–H5E⋯O6	0.90	2.15	2.965(3)	150
N5–H5E⋯O5	0.90	2.97	3.284(3)	102
N5–H5D⋯O17^ii^	0.90	2.44	3.310(3)	163
N8–H8E⋯O19	0.86	2.12	2.819(3)	138
N8–H8D⋯O10^iii^	0.86	2.83	3.470(3)	132
N8–H8D⋯O9^iii^	0.86	2.18	3.038(3)	176
N4–H4D⋯O22^iv^	0.86	1.99	2.775(4)	150
N4–H4E⋯O11^iv^	0.86	2.20	3.049(3)	170
N4–H4E⋯O12^iv^	0.86	2.68	3.351(3)	136
N3–H3D⋯O12^iv^	0.86	1.95	2.796(3)	166
N3–H3E⋯O14^i^	0.86	2.15	2.980(3)	162
N3–H3E⋯O18^v^	0.86	2.88	3.491(4)	130
N12–H12D⋯O4^iv^	0.86	2.00	2.840(3)	166
N12–H12E⋯O5^vi^	0.86	2.00	2.851(4)	168
N11–H11D⋯O3^iv^	0.86	2.25	3.094(3)	169
N11–H11D⋯O4^iv^	0.86	2.74	3.409(3)	135
N11–H11E⋯O21^vii^	0.86	2.11	2.830(3)	141
N10–H10D⋯O6^vi^	0.86	2.17	2.985(3)	157
N10–H10D⋯O5^vi^	0.86	2.71	3.406(3)	139
N13–H13D⋯O18^iii^	0.90	2.56	3.212(3)	130
N13–H13D⋯O19^viii^	0.90	2.38	3.255(3)	163
N15–H15E⋯O2^iii^	0.86	2.81	3.422(3)	130
N15–H15E⋯O1^iii^	0.86	2.11	2.973(3)	175
N15–H15D⋯O17^ix^	0.86	2.06	2.778(3)	140
N16–H16E⋯O2^iii^	0.86	1.99	2.791(3)	154
N16–H16D⋯O8^ix^	0.86	2.09	2.904(3)	158
N7–H7E⋯O10^iii^	0.86	1.98	2.805(3)	160
N7–H7D⋯O16^x^	0.86	2.14	2.928(3)	152
N14–H14D⋯O7^ix^	0.86	2.01	2.863(3)	168
N2–H2E⋯O13^i^	0.86	1.99	2.844(3)	170
N6–H6D⋯O16^x^	0.86	2.74	3.416(3)	137
N6–H6D⋯O15^x^	0.86	2.05	2.903(3)	171
O20–H8W⋯O8	0.86	1.86	2.721(2)	178
O20–H7W⋯O5^ix^	0.80	1.92	2.673(2)	155
O21–H10W⋯O13	1.00	1.79	2.710(3)	150
O21–H9W⋯O20	0.91	1.87	2.773(3)	174.4
O21–H9W⋯N1^ix^	0.91	2.97	3.339(3)	106.2
O22–H11W⋯O14	0.92	1.90	2.819(3)	171.7
O22–H12W⋯O6^vi^	0.78	1.96	2.726(3)	167
O18–H3W⋯O16^viii^	0.81	1.90	2.666(3)	157
O18–H4W⋯O14^ii^	0.91	1.87	2.771(2)	170
O19–H5W⋯O7^xi^	0.73	2.04	2.759(3)	172
O19–H6W⋯O18^xii^	0.77	1.94	2.696(3)	166
O17–H2W⋯O15^xiii^	0.84	1.94	2.715(3)	154
O17–H1W⋯O8^xi^	0.83	2.06	2.821(3)	154

a Symmetry codes: (I): (i) *x* − 1, *y*, *z*; (ii) −*x* + 2, *y* + 1/2, −*z* + 2; (iii) −*x* + 2, *y* − 1/2, −*z* + 2; (iv) −*x* + 2, *y* + 1/2, −*z* + 3; (v) −*x* + 3, *y* − 1/2, −*z* + 3. (II): (i) *x* + 1, *y*, *z*; (ii) −*x* + 1, *y* + 1/2, −*z* + 1; (iii) −*x*, *y* − 1/2, −*z* + 1; (iv) −*x* + 2, *y* + 1/2, −*z* + 2; (v) −*x* + 2, *y* − 1/2, −*z* + 1; (vi) *x*, *y*, *z* + 1; (vii) −*x* + 1, *y* + 1/2, −*z* + 2; (viii) −*x*, *y* + 1/2, −*z* + 1; (ix) *x* − 1, *y*, *z*; (x) *x*, *y*, *z* − 1; (xi) −*x* + 1, *y* − 1/2, −*z* + 1; (xii) *x*, *y* − 1, *z*; (xiii) −*x* + 1, *y* − 1/2, −*z* + 2.

### 3.2. Hirshfeld surface analysis

**Fig. 3:**
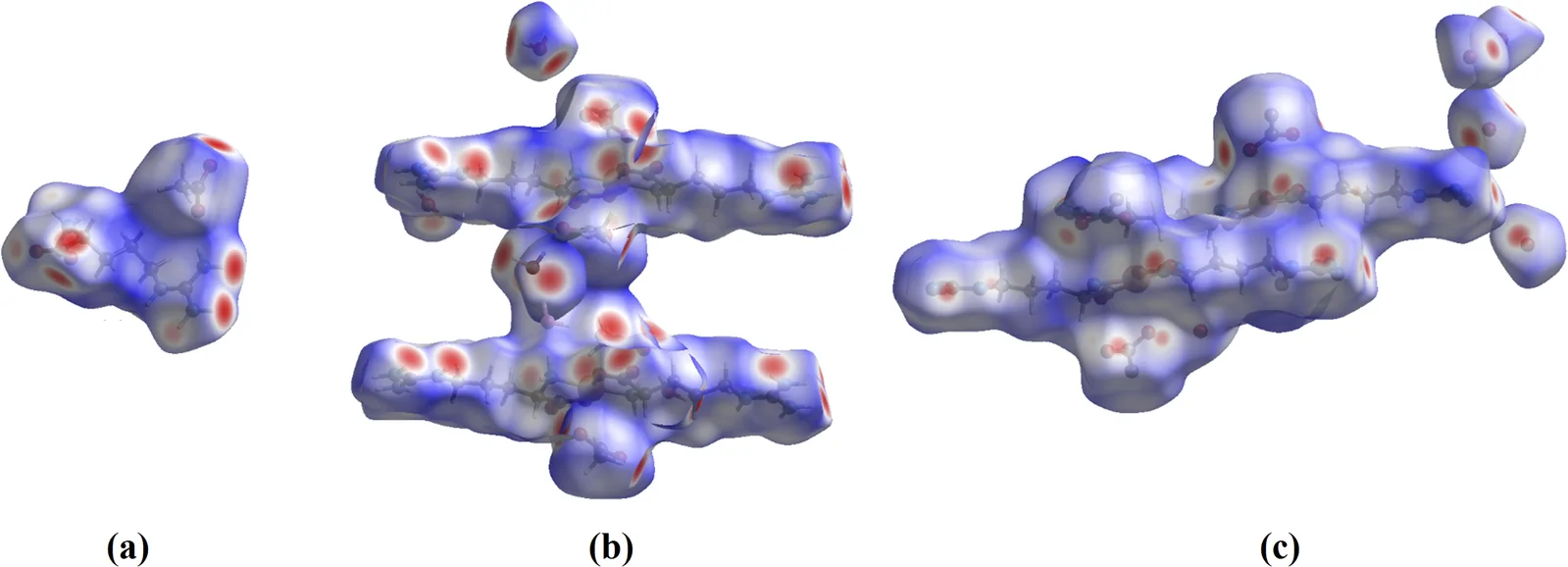
Hirshfeld surfaces mapped over *d*_norm_ for the studied structures (a) (I), (b) (II) and (c) DUYCAB.

For a better understanding of the supramolecular non-covalent interactions in the studied systems, a comparative Hirshfeld surface (HS) analysis was performed to visualize and quantify the differences and/or similarities of the intermolecular contacts across the crystal structures of (I), (II) and DUYCAB. The surfaces mapped over *d*_norm_, for (I), (II) and DUYCAB, plotted in the respective ranges [−0.617 (red), 1.370 (blue)], [−0.676 (red), 1.356 (blue)] and [−0.254 (red), 1.690 (blue)] are displayed in Fig. 3.

The presence of faint dark-red spots on the HS of (I) highlights the importance of the N–H⋯O interactions in stabilizing the crystal packing. Furthermore, the molecular HS generated for (II) and DUYCAB exhibited distinctly different shapes, reflecting therefore the different packing modes of the two complexes. In both complexes, each a.u. participates in numerous H-bonding contacts, which necessarily dominate both crystal structures. For instance, the guanidine groups are implicated in four hydrogen bonds in (II) and three H-bonds in the structure of DUYCAB. In addition, the oxygen atoms of the carboxylate groups display two large red spots in (II), while DUYCAB shows only one faint red spot. Also, it is worth noting that (II) exhibits multiple large dark red spots in comparison to the DUYCAB structure.

**Fig. 4:**
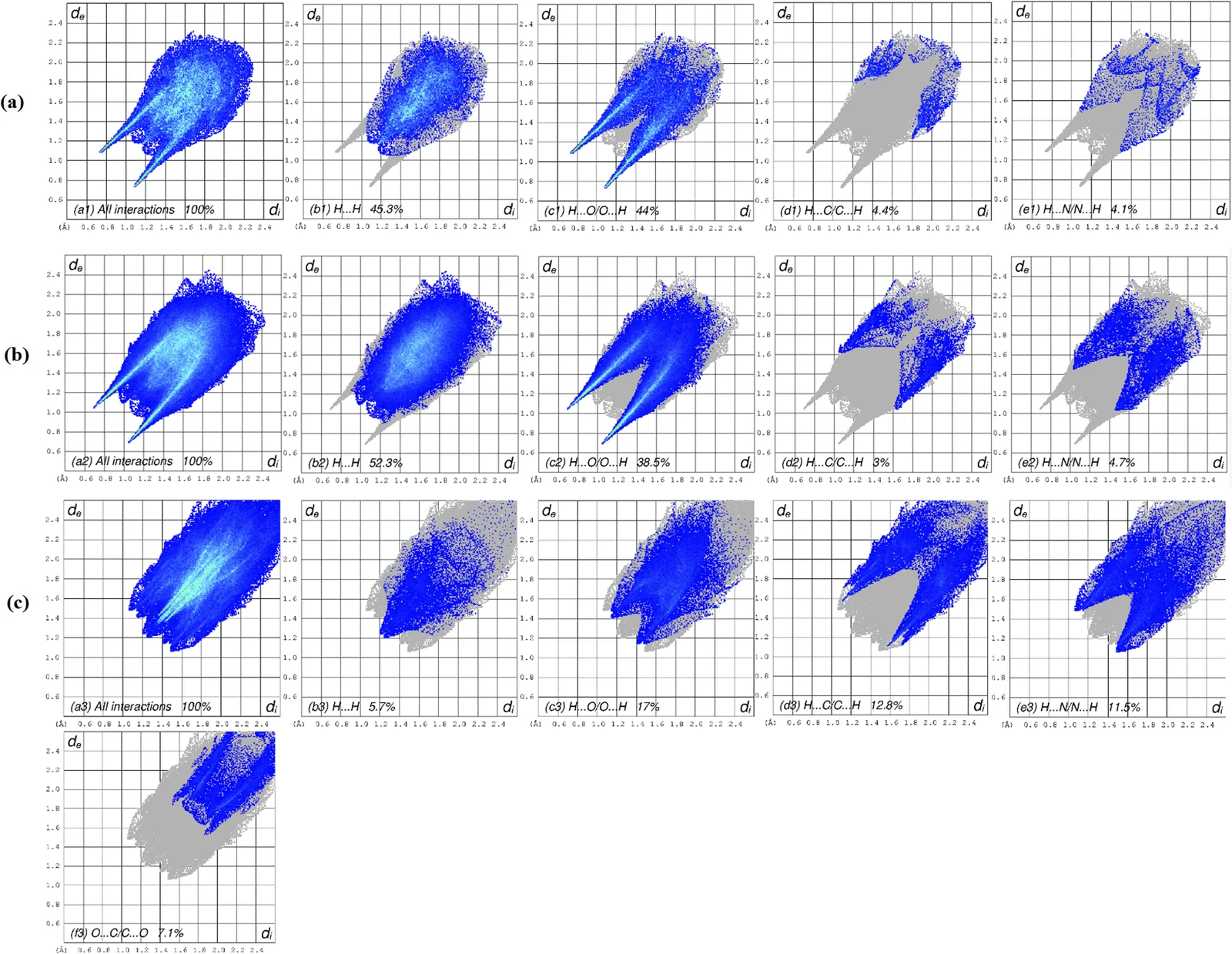
2D-FP plots showing the most prominent contacts in (a) (I), (b) (II) and (c) DUYCAB.

The quantitative contribution of the intermolecular contacts governing the crystal packing of the analyzed structures was evaluated by drawing the two-dimensional fingerprint (FP) plots, which provide a detailed comparison of the supramolecular environments and reveal distinct packing characteristics for each structure. The overall 2D-FP plots (Fig. 4) exhibit different silhouettes, reflecting variations in the intermolecular interaction patterns among the two complexes. In both (I) and (II), the H⋯H contacts showed predominant contributions, accounting for 45.3% and 52.3%, respectively. Such predominance is characteristic of hydrogen-rich organic compounds, highlighting the contribution of dispersive forces to the crystal stability. The second most significant contribution arises from the H⋯O/O⋯H contacts, which reach 44.0% in (I) and 38.5% in (II), underlining the importance of extensive hydrogen-bonding networks involving oxygen atoms. In addition, the H⋯C/C⋯H and H⋯N/N⋯H contacts display a less important contribution to the packing, with the respective values of 4.4% and 4.1% for (I), and 3.0% and 4.7% for (II). These contacts were found to complement the dominant H-bonding interactions and contribute to the overall cohesion of the studied crystal structures.

In contrast, the structure of DUYCAB displays a notably different intermolecular interactions' profile, despite its structural similarity to (II). In this case, the H⋯H and H⋯O/O⋯H contacts contribute only with the respective percentages 5.7% and 17.0%, indicating a reduced influence of the classical H-bonding and van der Waals interactions. Instead, the H⋯C/C⋯H (12.8%) and H⋯N/N⋯H (11.5%) contacts are significantly more prominent, suggesting a reorganization of the intermolecular contacts in DUYCAB compared to the two other structures.

**Fig. 5:**
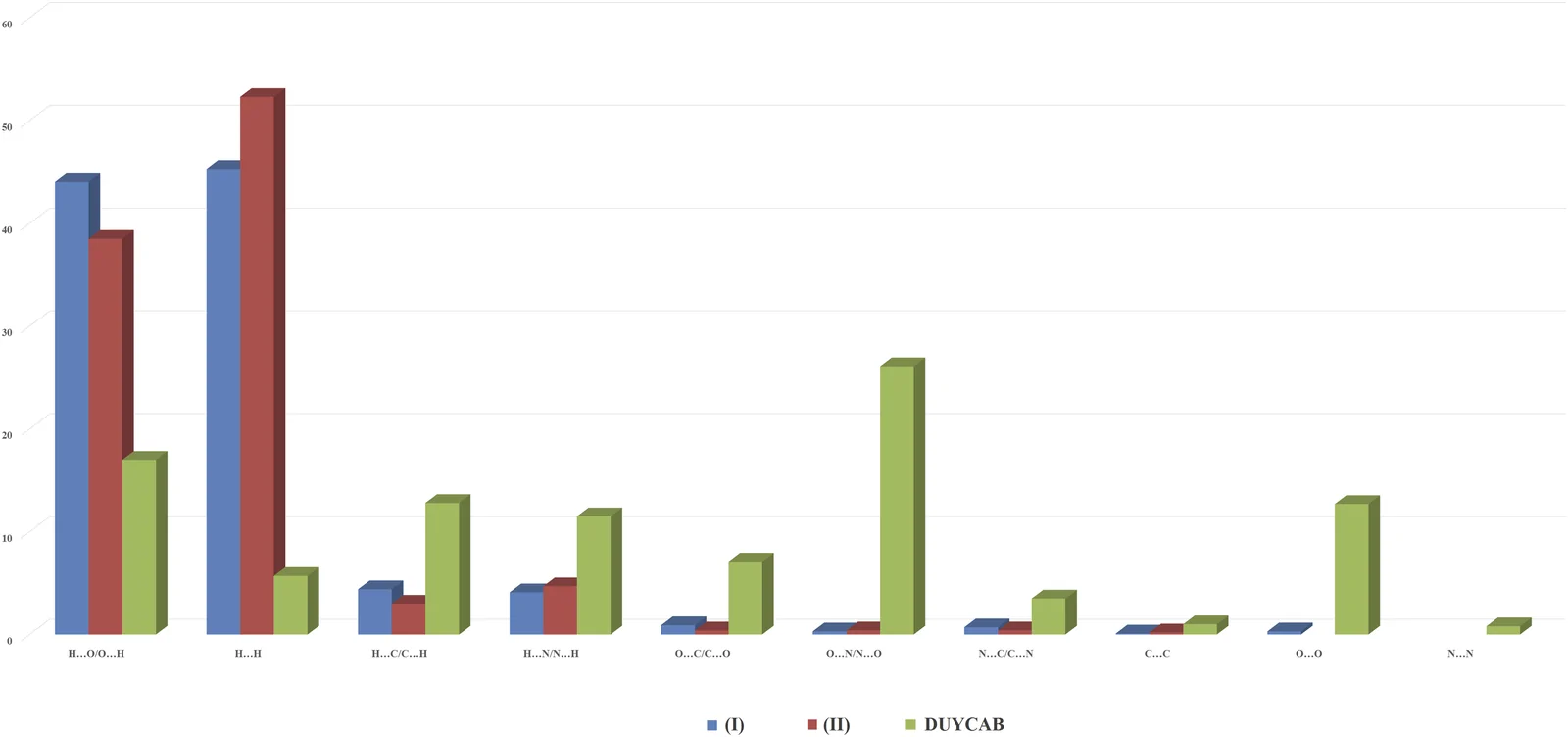
Percentage contributions of the different contacts to the overall HSs in the studied structures.

Notably, the oxygen and nitrogen atoms contribute predominantly to the DUYCAB packing, by forming O⋯N/N⋯O contacts that count for 26.1%. It is worth noting that these contacts were found to be negligible (<1%) in both (I) and (II). Furthermore, the O⋯C/C⋯O (7.1%) and O⋯O (12.7%) contacts are observed exclusively in DUYCAB, and are essentially absent in (I) and (II). This pronounced contribution of oxygen-involving contacts highlights a fundamentally different supramolecular organization driven by different interactions. These results clearly demonstrate that while (I) and (II) are primarily stabilized by hydrogen-dominated interactions, DUYCAB adopts a packing motif governed by strong heteroatom⋯heteroatom contacts, leading to distinct crystal architectures, as shown in Fig. 5. The detailed contacts' contributions in the structures of (I), (II) and DUYCAB are given in Fig. S1–S3.

### 3.3. DFT calculations

Geometry optimizations and electronic structure calculations were performed using the B3LYP hybrid density functional together with the LANL2DZ basis set. The B3LYP functional is a widely used hybrid GGA density functional that has consistently demonstrated reliable performance for the structural and electronic characterization of transition-metal complexes. In combination with the LANL2DZ basis set, which incorporates an effective core potential (ECP) for transition metals, it provides a good balance between computational efficiency and accuracy for Cu-containing systems, while maintaining a reasonable computational cost.

Recent benchmark and application studies have further demonstrated the suitability of the B3LYP/LANL2DZ approach for transition metal-systems,^38,39^ by reproducing experimental metal–ligand bond properties with good accuracy. For example, Kosar *et al.*, reported that it produced one of the smallest deviations in Cu–O bond dissociation energies among the functionals evaluated, supporting its reliability for copper complexes.^38^ Furthermore, recent applications of the same computational protocol have successfully employed B3LYP/LANL2DZ for the geometry optimization, electronic structure analysis and stability evaluation of Cu complexes, demonstrating that this methodology remains an accepted and reliable choice for transition-metal systems.^39^

#### 3.3.1. Molecular electrostatic potential analysis

**Fig. 6:**
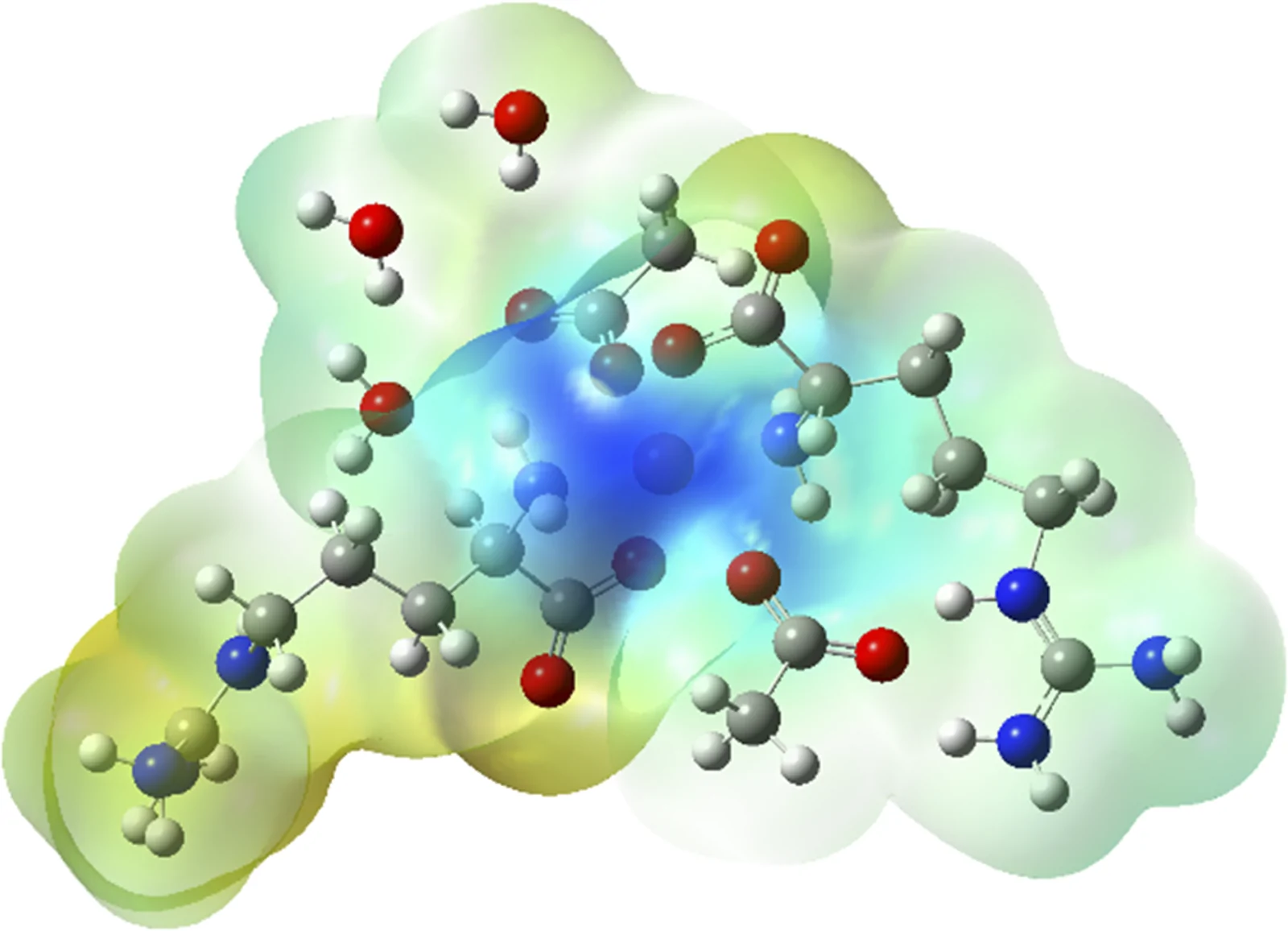
MEP surface plotted for complex (II).

Molecular electrostatic potential (MEP) and electrostatic interactions of (II) were investigated using the same theoretical framework as that used for the optimization. The MEP surface provides the necessary information about the most reactive sites in a molecular system. The values of the electrostatic potential at the surface are shown in five different colors. The color-coded surface indicates that the potential increases in the following order: red < green < blue. The most negatively charged regions are characterized by red coloration, marking the most favored sites for a possible electrophilic attack. The most positive value, which appears in blue, indicates the sites that are most preferred for a nucleophilic attack. The MEP area displayed in Fig. 6 indicates that the most negative potential is focused around the oxygen atoms of the l-arginine ligands and the acetate counter-ions in complex (II), suggesting therefore the best sites for a possible electrophilic attack. Furthermore, the green regions are concentrated around the nitrogen atoms and show a neutral potential, while a positive potential is found around the copper at the heart of the molecule and on the external hydrogen atoms of the ligand units.

#### 3.3.2. Reactivity descriptors

The global reactivity descriptors were derived from the frontier molecular orbital (FMO) energies, specifically the highest occupied molecular orbital (HOMO) and the lowest unoccupied molecular orbital (LUMO). The relatively high ionization energy (*I* = −*E*_HOMO_ = 2.915 eV) and negative chemical potential (*µ* = −2.899 eV) indicate the intrinsic stability of compound (II), suggesting a low tendency to decompose into its constituent components. The low chemical hardness (*η* = 0.040 eV) reflects the ease of the electron cloud deformations under small perturbations. In addition, the very small HOMO–LUMO energy gap (Δ*E* = 0.039 eV), derived from the ionization energy (*I* = −*E*_HOMO_ = 2.915 eV) and electron affinity (*A* = −*E*_LUMO_ = 2.876 eV), corresponds to a high chemical softness (*S* = 25.66 eV^−1^). Therefore, this indicates an enhanced chemical flexibility and a potentially high chemical and biological reactivity. The large electrophilicity index (*ω* = 107.6 eV) further demonstrates the strong electrophilic character of compound (II). However, it must be acknowledged that for open-shell transition metal complexes like the present d^9^ Cu(ii) system, Koopmans' theorem is often quantitatively unreliable due to significant electronic relaxation effects and strong electron correlation within the partially filled 3d subshell. Consequently, these derived indices are interpreted herein as qualitative electronic descriptors reflecting structural trends rather than absolute thermodynamic values.

**Fig. 7:**
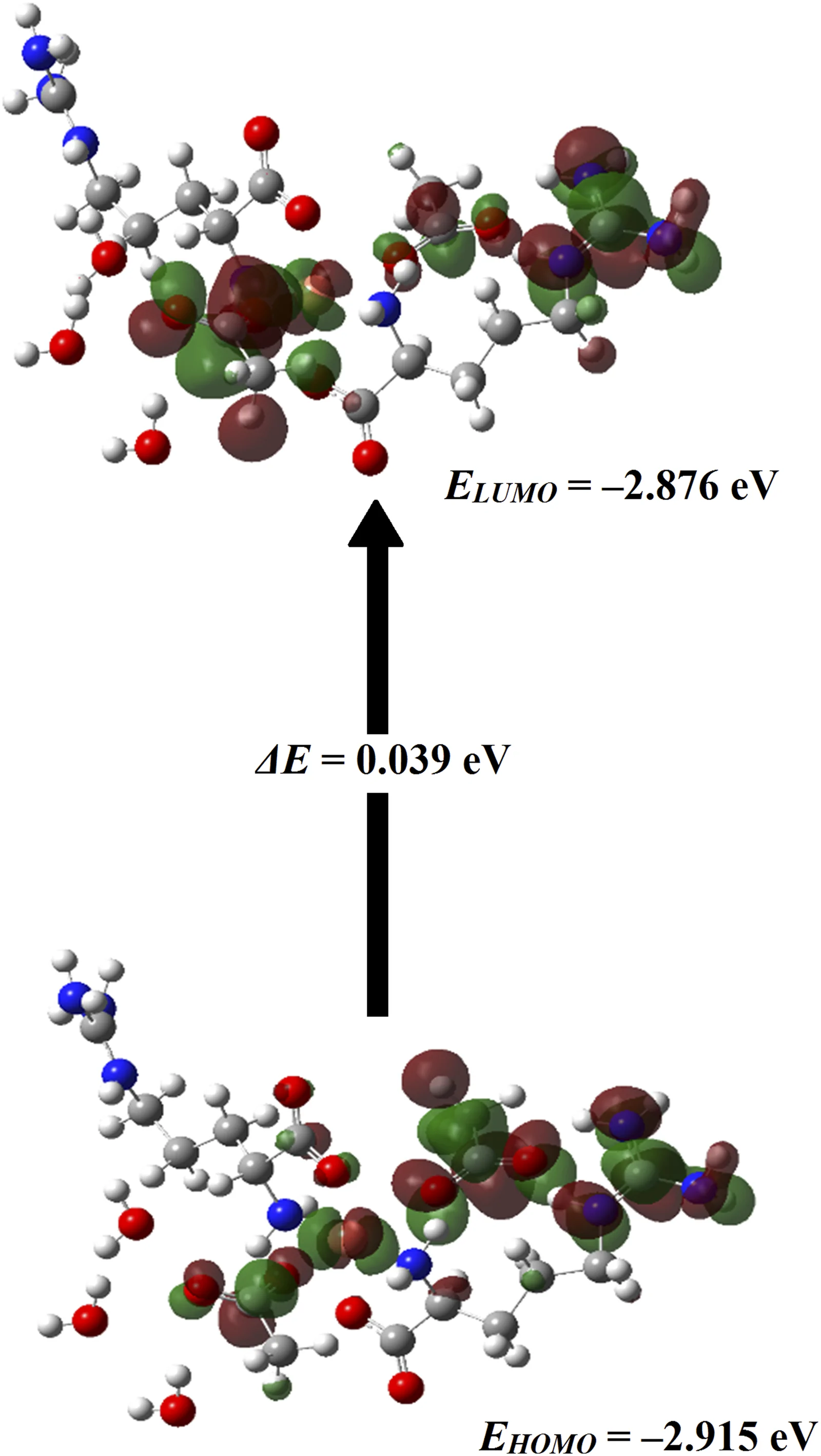
Schematic presentation of the FMOs computed for complex (II).

In the neutral state, both the HOMO and LUMO are predominantly associated with the Cu(ii) center. The HOMO is mainly localized over the guanidinium moiety, acetate anion, and carboxylate group of another acetate ligand. However, the LUMO is delocalized over both acetate groups, the guanidinium moiety of one arginine ligand, and amino group of the second ligand (Fig. 7). The narrow HOMO–LUMO gap indicates a high chemical reactivity and relatively a low kinetic stability of complex (II), facilitating possible intramolecular charge transfer processes.

#### 3.3.3. QTAIM analyses

**Fig. 8:**
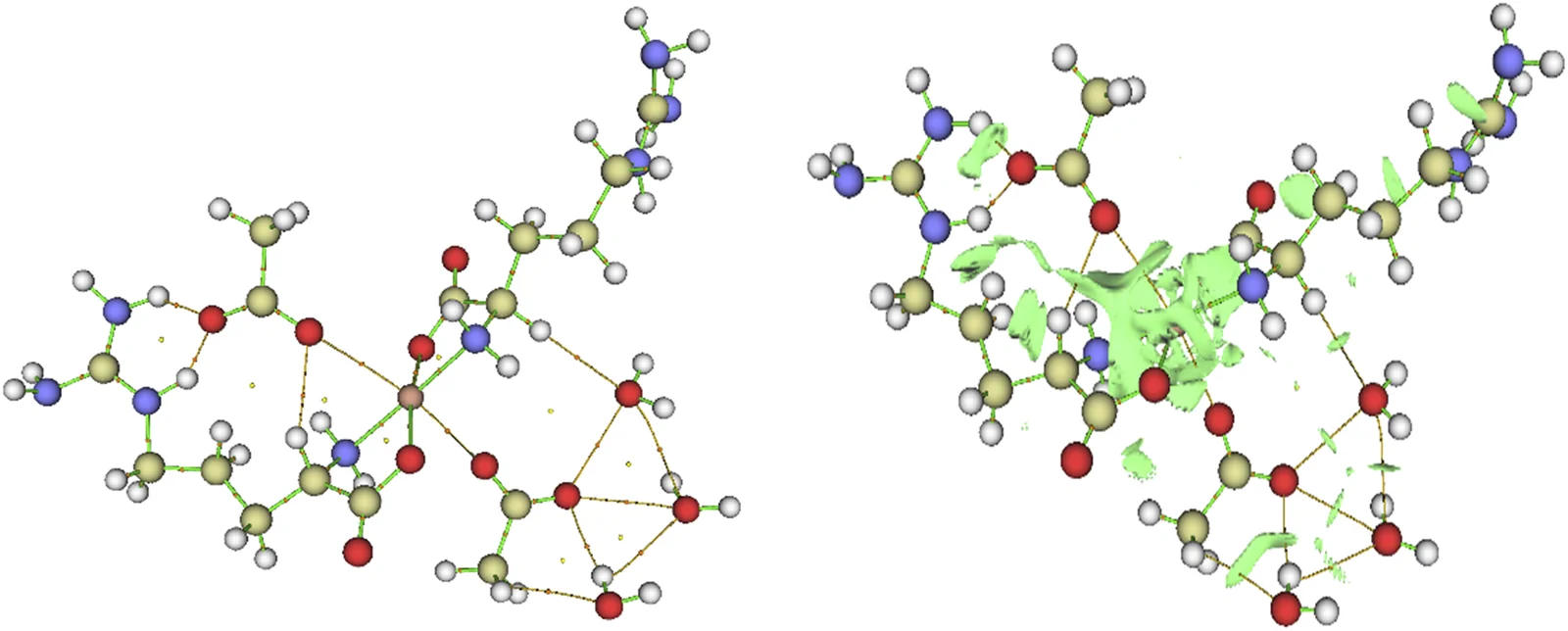
QTAIM surface maps computed for (II).

QTAIM approach is a significant theory for examining and characterizing chemical connections. Characteristics associated with CP (3, −1) and electron density's assessment provide crucial insights into the chemical bonds occurring in the studied complex. QTAIM analysis was conducted to identify and evaluate all the essential bonding locations. The molecular representation of the systems is illustrated in Fig. 8. The topological parameters were extracted using Bader's AIM framework,^28^ which includes the electron density *ρ*(*r*), density Laplacian ∇^2^(*r*), potential energy density *V*(*r*), kinetic energy density *G*(*r*) and total electron energy *H*(*r*). The parameters *ρ*(*r*) and *H*(*r*), along with ∇^2^(*r*), showed negative values at the significant O–Cu bonding sites, suggesting strong coordination bonds. Moreover, we have observed relatively low values of the electron density *ρ*(*r*), positive values of the Laplacian ∇^2^(*r*), and negative values for the total electron energy *H*(*r*). According to the classification established by Espinosa*et al.*,^40^ these indicators represent weak intermediate-type connections. Based on the obtained results, (|*V*(*r*)|/*G*(*r*)| > 2, ∇^2^(*r*) < 0, and *H*(*r*) < 0), the bond is fully covalent, where the electron potential energy density *V*(*r*) is locally predominant in the interatomic area. Also, the bond is classified as non-covalent if 1 < |*V*(*r*)|/*G*(*r*)| < 2, ∇^2^(*r*) > 0, and *H*(*r*) < 0.

#### 3.3.4. ELF and LOL maps

**Fig. 9:**
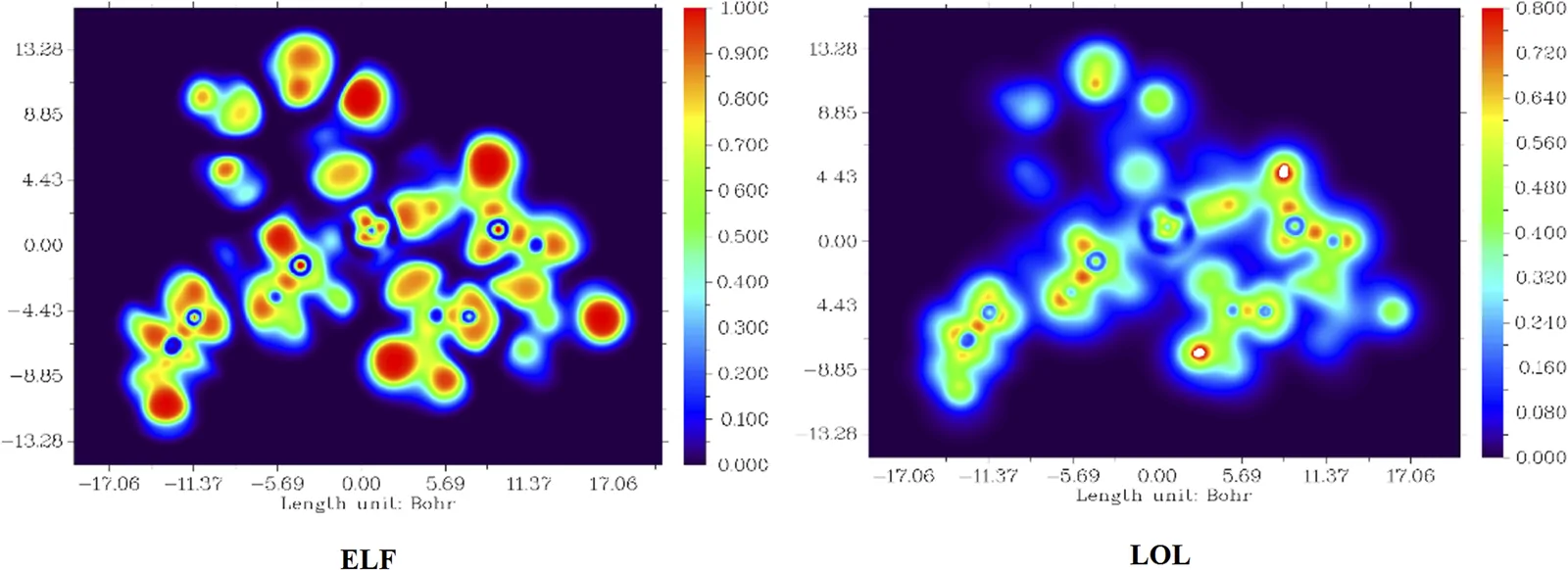
ELF and LOL maps computed for complex (II).

Examining the electronic localization function (ELF) and the localized orbital localizer (LOL) maps reveals important details about the surface of the topological maps.^31,40,41^ The evaluation of ELF and LOL allowed us to identify the electron pairs on the molecules' surfaces in (II). Fig. 9 highlights the findings, where the larger or smaller peak areas illustrate the electronic environment of each atom. The LOL shows the maximum localized orbitals that overlap because of the orbital gradient, while the ELF shows the concentration of the electron pairs. Furthermore, the ELF map ranges from 0.0 to 1.0, but values lower than 0.5 indicate areas with electron displacement. In contrast, the LOL shows high values bigger than 0.5 in areas where the electron density is defined by the electrons' localization.

The colors in the ELF and LOL maps represent the locations of the bonding and unbonded electrons. Additionally, the red areas around the hydrogen atoms, which have the highest values, show both bonded pairs of electrons in addition to unbonded electrons. The elevated values of LOL or ELF, marked in red around the hydrogen atoms, signify strong electron localization due to the presence of covalent bonds, unpaired electrons, or nuclear shells in those areas. The hydrogen atoms' centers in the LOL representation is colored in white, because the electron density exceeds the highest point of the color scale. As illustrated in Fig. 9, the carbon and nitrogen atoms are surrounded by blue areas that indicate a lack of electrons, and the red area around the hydrogens was also indicated.

#### 3.3.5. NCI-RDG analysis

**Fig. 10:**
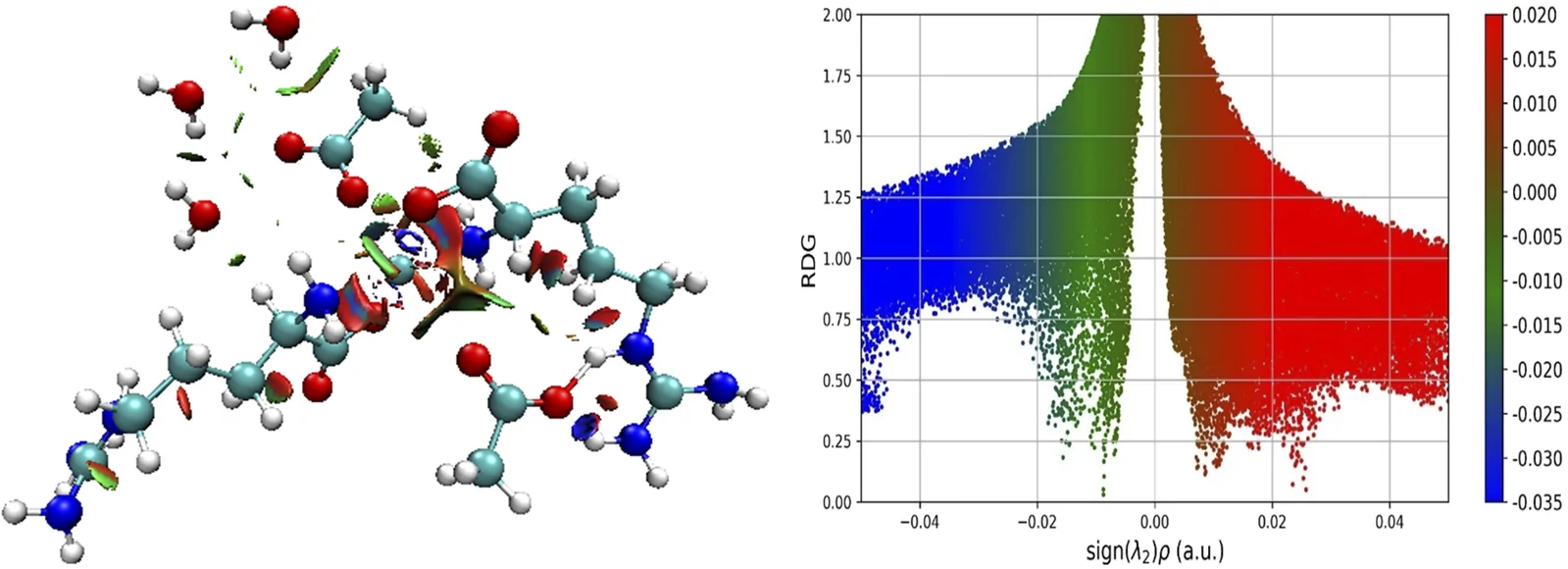
NCI (left) and RDG (right) plots obtained for complex (II).

The color/contour combination of the non-covalent interaction index (NCI) and reduced density gradient (RDG) analysis (NCI-RDG), alongside the scatter plot for complex (II) are shown in Fig. 10. The two-dimensional scatter plots obtained for (II) depend on the sign(*λ*_2_)*ρ*(*r*) included in the same figure, showing distinct non-covalent interaction models in the molecule. For *λ*_2_ values higher than 0, this indicates non-bonding and strongly repulsive forces, resulting in steric effects within the coordination sphere (shown in red), while *λ*_2_ values smaller than 0 indicate attracting forces and strong bonding interactions (shown in blue). Furthermore, a zero value of *λ*_2_ indicates areas of weak interactions, such as van der Waals forces and π–π stacking, which are represented in green with values between −0.02 and 0.01 atomic units (a.u.) (see Fig. 10). The RDG graphs reveal that the hydrogen interactions are in the range of [−0.05, −0.02] a.u., and that the range of 0.01 to 0.05 a.u. is associated with steric effects in the coordination sphere.^31^

### 3.4. Antimicrobial tests

The antimicrobial effectiveness of compounds (I) and (II) was evaluated in terms of the zones of inhibition (ZOI) in mm, by comparing their values against those of standard reference drugs; Chloramphenicol (Chl) and Nystalin (Nyst). The results of the microbial assays are given in Table 3.

**Table 3:** Inhibition zones (mm) and MIC values (mg mL^−1^) of tested microorganisms at different concentrations (C1 = 20, C2 = 10, C3 = 5 and C4 = 2.5 mg mL^−1^) of (I) and (II)^a^

Tested micro-organism	Zones of inhibition											
	Studied compounds										Standard drugs	
	(I)					(II)					Chl	Nyst
	C1	C2	C3	C4	MIC	C1	C2	C3	C4	MIC		
*Staphylococcus aureus*	15.0 ± 0.5	13.5 ± 0.5	12.0 ± 0.5	10.0 ± 0.0	1.25	30.0 ± 0.0	23.0 ± 0.5	20.5 ± 0.5	17.0 ± 0.5	0.625	30.0 ± 0.5	—
*Escherichia coli*	15.5 ± 0.5	10.0 ± 0.5	—	—	10	16.0 ± 0.5	12.5 ± 0.5	11.0 ± 0.0	9.5 ± 0.5	1.25	24.5 ± 0.5	—
*Candida albicans*	—	—	—	—	—	17.0 ± 0.5	15.0 ± 0.0	10.0 ± 0.5	8.5 ± 0.5	1.25	—	33.0 ± 0.0
*Aspergillus niger*	—	—	—	—	—	14.5 ± 0.5	11.0 ± 0.5	9.5 ± 0.5	6.5 ± 0.5	2.5	—	19.5 ± 0.5

a The values represent the mean ± absolute deviation of independent duplicate runs.

Compound (II) exhibited a pronounced antibacterial efficacy against the Gram-positive *S. aureus*, producing a highly active ZOI of 30.0 ± 0.0 mm at 20 mg mL^−1^, thus matching the potency of the standard drug (Chl). In contrast, (I) is half effective as (II) at the same concentration, showing a ZOI of only 15.0 ± 0.5 mm. As for the Gram-negative *E. coli*, while (I) exhibited a samilar behavior as against the former strain (with a ZOI value of 15.5 ± 0.5 mm at 20 mg mL^−1^), compound (II) showed a significant drop in efficacy (ZOI of 16.0 ± 0.5 mm) at the same concentration (20 mg mL^−1^) compared to its performance against *S. aureus* and also the drug's area of inhibition being 24.5 ± 0.5 mm.

Moreover, even when diluted down to the lowest tested concentration (2.5 mg mL^−1^), complex (II) demonstrated remarkably strong inhibitory behavior, maintaining a highly active clearing zone of ≈17.0 ± 0.5 mm against *S. aureus* (and 9.5 ± 0.5 against *E. coli*), strongly suggesting that its true threshold of antimicrobial efficacy extended far below this boundary. This was subsequently confirmed *via* extended broth microdilution assays starting from 2.5 mg mL^−1^, which revealed visible bacterial growth inhibition and true absolute Minimum Inhibitory Concentration (MIC) values of 625 µg mL^−1^ and 1.25 mg mL^−1^ against *S. aureus* and *E. coli*, respectively. These values demonstrate robust bactericidal activity of (II) and its highly potent profile aligns closely with structurally related copper–arginine frameworks found in the literature. For instance, the benchmarks established by Wojciechowska *et al.*,^42^ for their binary copper–arginine oxalate complex ([Cu(l-Arg)_2_(H_2_O)]C_2_O_4_·6H_2_O) showing MIC thresholds of 0.25 to 0.50 mg mL^−1^, directly mirrors our findings.

Furthermore, (II), exhibited moderate antifungal activity against *C. albicans*, with a ZOI value of 17.0 ± 0.5 mm at 20 mg mL^−1^. This value remains less significant than the standard's (Nyst), which yielded an inhibition area of 33.0 ± 0.0 mm. Also, it is worth noting that both (II) and (Nyst) showed similar strong inhibition activity against *A. niger*, with the related ZOI values being 14.5 ± 0.5 mm (at 20 mg mL^−1^) and 19.5 ± 0.5 mm, respectively. Interestingly, while the complex (II) displayed a relatively narrow ZOI of <10 mm at the lowest tested concentration (2.5 mg mL^−1^) against *C. albicans* and *A. niger*, it exhibited a strong antifungal potency by inhibiting visible growth across both the strains, and demonstrated a distinct MIC down to value of 1.25 mg mL^−1^ in broth macro-dilution for *C. albicans* and 2.5 mg mL^−1^ for *A. niger*. However, (I) was completely inactive against both fungal strains across all tested concentrations (C1–C4).

Notably, these results confirm the viability of (II) as a powerful broad-spectrum antimicrobial candidate, with an order of sensitivity against the four tested strains being: *S. aureus* > *C. albicans*, *E. coli* > *A. niger*. Whereas the activity of (I) was found to be strictly antibacterial, by showing equal sensitivity for both *S. aureus* and *E. coli* at 20 mg mL^−1^.

## 4. Conclusions

In this search, l-argininium acetate (compound I) and its copper(ii) complex, acetatobis(l-arginine)copper(ii) acetate trihydrate [Cu(OAc)(Arg)_2_](OAc)·3H_2_O (compound II), were successfully synthesized and structurally characterized. Single-crystal X-ray diffraction analysis revealed that both compounds crystallize in the monoclinic space group *P*2_1_, with the copper(ii) center in compound (II) adopting a distorted square-pyramidal coordination geometry involving nitrogen and oxygen donor atoms from l-arginine ligands and acetate groups. A dense three-dimensional hydrogen-bonding network involving guanidinium groups, acetate anions, and lattice water molecules was found to play a crucial role in stabilizing the crystal packing. Moreover, the HS and FP analyses confirmed that the H⋯H and H⋯O/O⋯H contacts dominate the intermolecular interactions, highlighting the importance of hydrogen-bonding interactions and non-conventional forces in maintaining the structural cohesion of the crystal lattice.

Theoretical investigations, including DFT-based reactivity descriptors, molecular electrostatic potential, ELF/LOL, and QTAIM analyses, provided deeper insight into the electronic structure and bonding characteristics of the copper complex, confirming the presence of strong coordination bonds and favorable charge distribution patterns that support structural stability and reactivity.

Furthermore, the *in vitro* antimicrobial assays demonstrated that compound (II) exhibits enhanced inhibitory activity compared to its precursor ligand (I), indicating that the coordination of l-arginine to the copper(ii) center significantly improves the biological performance of the system. This enhancement can be attributed to the synergistic effect between the metal center and the bioactive amino acid ligand.

In general, the combined structural, theoretical, and biological findings highlight the potential of arginine-based copper(ii) complexes as promising candidates for the development of new bioactive coordination compounds. The present study provides valuable insights into the structure–activity relationships of amino acid-derived metal complexes and establishes a solid foundation for further exploration of related systems with enhanced pharmacological potential.

## Consent for publication

All the authors read and approved this version of the manuscript.

## Author contributions

AD: conceptualization, writing – original draft, review & editing, resources, visualization, methodology, validation. SS: writing – original draft, resources, visualization, methodology. HA: writing – original draft, resources, visualization, methodology. MSMA: review & editing, resources, methodology, validation. CP: review & editing, resources, methodology, validation. OA: writing – original draft, resources, methodology. SGG: review & editing, resources, validation. PR: review & editing, resources, validation. RG: review & editing, resources, validation.

## Conflicts of interest

The authors declare that they have no known conflict of interest that could have appeared to influence the work reported in this paper. The funders had no role in the design of the study; in the collection, analyses, or interpretation of data; in the writing of the manuscript or in the decision to publish the results.

## Supplementary Material

RA-016-D6RA03860C-s001

RA-016-D6RA03860C-s002

## Data Availability

CCDC 2523709 (I) and 2523710 (II) contain the supplementary crystallographic data for this paper.^43*a*,*b*^ Data supporting this study are included within the article and its supplementary information (SI). Supplementary information: 2D-FP plots of the overall interactions in decomposed contacts' contribution in the structures of (I), (II) and DUYCAB. See DOI: https://doi.org/10.1039/d6ra03860c.

## References

[cit1] Machado F. L. A., Sousa L. L. L., Cunha R. O., Cabral F. A. O., Rodrigues A. R., Carvalho J. F., Santana R. C. (2010). Specific heat measurements in pure and in (Cu, Mn, Fe, Ni)-doped single-crystals of L-arginine phosphate monohydrate. J. Phys. Chem. Solids.

[cit2] Pragasam A. J. A., Selvakumar S., Thamizharasan K., Prem Anand D., Sagayaraj P. (2005). Growth and optical characterization of Cu-and Mg-substituted L-arginine di phosphate single crystals. J. Cryst. Growth.

[cit3] Prasanyaa T., Haris M., Jayaramakrishnan V., Amgalan M., Mathivanan V. (2013). Synthesis, characterization and anti-microbial activity of pure, Cu2+ and Cd2+ doped organic NLO L-arginine trifluoroacetate single crystals. Phys. Scr..

[cit4] Patra A. K., Bhowmick T., Ramakumar S., Chakravarty A. R. (2007). Inorg. Chem..

[cit5] Patra A. K., TuhinBhowmick S. R., Suryanarayanarao R., Chakravarty A. R. (2009). Copper (II) complexes of L-arginine as netropsin mimics showing DNA cleavage activity in red light. Inorg. Chem..

[cit6] Wojciechowska A., Gągor A., Zierkiewicz W., Jarząb A., Dylonga A., Duczmal M. (2015). Metal–organic framework in an l-arginine copper(ii) ion polymer: structure, properties, theoretical studies and microbiological activity. RSC Adv..

[cit7] Ngece K., Khwaza V., Paca A. M., Aderibigbe B. A. (2025). The Antimicrobial Efficacy of Copper Complexes: A Review. Antibiotics.

[cit8] Hemissi H., Nasri M., Abid S., Al-Deyab S. S., Dhahri E., Hlil E. K., Rzaigui M. (2012). Crystal structure, spectroscopic, magnetic and electronic structure studies of a novel Cu (II) amino acid complex [Cu(l-arg)2(H2O)]2(P4O12)·8H2O. J. Solid State Chem..

[cit9] Wojciechowska A., Kochel A., Duczmal M. (2016). Supramolecular assemblies in [Cu(L-Arg)2(H2O)]C2O4·6H2O complex–Structural, spectroscopic, magnetic and thermal behavior. Mater. Chem. Phys..

[cit10] Guang F., Ling-Juan D., Zhan-Ying M., Xiao-Bo L., Yin-Li Z., Jia-Juan S. (2016). Chin. J. Struct. Chem..

[cit11] Daryanavard M., Jarrah N. (2019). The inhibitory effect of the amino acid complexes of Zn (II) on the growth of Aspergillusflavus and aflatoxin B1 production. J. Iran. Chem. Soc..

[cit12] Hu R., Yu Q., Liang F., Ma L., Chen X., Zhang M., Liang H., Yu K. (2008). Syntheses and crystal structures of cis- and trans-copper (II) complexes of L-arginine. J. Coord. Chem..

[cit13] Alagha A., Brown D. A., Elawad M., Müller-Bunz H., Nimir H., Yanovsky A. I., Nolan K. B. (2011). The preparation and crystal structure of acetatobis (L-arginine) zinc (II) acetate trihydrate, the first reported X-ray structure of a zinc (II)–arginine complex. Inorg. Chim. Acta.

[cit14] Wojciechowska A., Janczak J., Rojek T., Gorzsas A., Malik-Gajewska M., Duczmal M. (2019). Isothiocyanate controlled architecture, spectroscopic, and magnetic behavior of copper (II) l–arginine complexes. J. Coord. Chem..

[cit15] Duarte M., Teresa Leal S., de CT Carrondo M. A. A. F., Simões Gonçalves M. L. S., Hursthouse M. B., Walker N. P. C., Dawes H. M. (1986). The preparation and crystal structure analysis of bis(L-Arginine)Cu(II) (acetate)2 trihydrate. Inorg. Chim. Acta.

[cit16] Laabassi M., Direm A., Athmani H., Hanfer M., Altomare A., Falcicchio A., De Feudis M., Boulcina R., Grée R. (2025). Unraveling the antioxidant effect of new quinoline epoxides: Synthesis, crystal structure from PXRD, HS analysis and molecular docking's binding modes. J. Mol. Struct..

[cit17] Khelaf R., Direm A., Hakkar F., Laabassi M., Boulcina R., Parlak C., Ramasami P. (2025). New Quinoline Derivatives as Potential Multitherapeutic Agents: A Facile Synthesis via an Epoxide Ring Opening Reaction, Characterization, DFT Calculations, and Molecular Docking. ChemistrySelect.

[cit18] Direm A., Parlak C., El Bali B., Abdelbaky M. S. M., Garcia-Granda S. Experimental and computational insights into polymorphism in an antimicrobial sulfadrug: discovery of a novel monoclinic form of sulfamerazine. 2024. J. Iran. Chem. Soc..

[cit19] El Bali B., Direm A., Lachkar M., Díaz-García D., Gómez-Ruiz S., Dihazi H. (2024). In vitro and in silico studies of a di-copper cyclam complex for anticancer application: functionalization, cytotoxicity, ADMET profile and molecular docking as a VEGFR1 inhibitor. Transition Met. Chem..

[cit20] Burla M. C., Caliandro R., Carrozzini B., Cascarano G. L., Cuocci C., Giacovazzo C., Mallamo M., Mazzone A., Polidori G. (2015). Crystal structure determination and refinement via SIR2014. J. Appl. Cryst..

[cit21] Sheldrick G. M. , SHELXL-2014/7: A Program for Structure Refinement, University of Göttingen, Göttingen, Germany, 2014

[cit22] Macrae C. F., Edgington P. R., McCabe P., Pidcock E., Shields G. P., Taylor R., Towler M., van de Streek J. (2006). Mercury : visualization and analysis of crystal structures. J. Appl. Crystallogr..

[cit23] Wolff S. K. , Grimwood D. J., McKinnon J. J., Turner M. J., Jayatilaka D. and Spackman M. A., Crystal Explorer, 2012

[cit24] Dennington R. D. , Keith T. A. and Millam J. M., GaussView 5.0.8, Gaussian Inc, 2009

[cit25] Samai S., Rouichi S., Ferhati A., Chakir A. (2016). N,N-dimethylformamide (DMF), and N,N-dimethylacetamide (DMA) reactions with NO3, OH and Cl: A theoretical study of the kinetics and mechanisms. Arabian J. Chem..

[cit26] Marcus D. H. , Curtis D. E., Lonie D. C., Vandermeersch T., Zurek E. and Hutchison G. R., Avogadro: Version 1.1.1, 2012 10.1186/1758-2946-4-17 PMC3542060 22889332

[cit27] Scrocco E., Tomasi J. (1978). Electronic Molecular Structure, Reactivity and Intermolecular Forces: An Euristic Interpretation by Means of Electrostatic Molecular Potentials. Adv. Quantum Chem..

[cit28] Zevallos J., Toro-Labbe A. (2003). A theoretical analysis of the Kohn-Sham and Hartree-Fock orbitals and their use in the determination of electronic properties. J. Chil. Chem. Soc..

[cit29] Lu T., Chen F. (2012). Multiwfn: a multifunctional Wavefunction analyzer. J. Comput. Chem..

[cit30] Humphrey W., Dalke A., Schulten K. (1996). VMD - visual molecu- lar dynamics. J. Mol. Graphics.

[cit31] Samai S., Direm A., Parlak C. (2024). Pyrazinamide-based Co(II), Ni(II) and Cu(II) complexes: DFT exploration of structure, reactivity properties and in silico ADMET profile. J. Mol. Model..

[cit32] Das R., Nag S., Banerjee P. (2023). Molecules.

[cit33] Parr R. G., Donnelly R. A., Levy M., Palke W. E. (2008). J. Chem. Phys..

[cit34] Parr R. G., Szentpály L. v., Liu S. (1999). J. Am. Chem. Soc..

[cit35] Chermette H. (1999). J. Comput. Chem..

[cit36] Ekong U. S., Mgbor N. C., Moneke A. N., Obi S. K. C. (2004). Evaluation of the antimicrobial and some pharmacokinetic properties of an antibiotic substance produced by an environmental Aspergillus species SK2 isolated from Nigerian soil. Niger. J. Microbiol..

[cit37] Okeke M. I., Iroegbu C. U., Eze E. N., Okoli A. S., Esimone C. O. (2001). Evaluation of extracts of the root of Landolphiaowerrience for antibacterial activity. J. Ethnopharmacol..

[cit38] Kosar N., Ayub K., Amjad Gilani M., Muhammad S., Mahmood T. Benchmark Density Functional Theory Approach for the Calculation of Bond Dissociation Energies of the M−O2 Bond: A Key Step in Water Splitting Reactions. (2022). ACS Omega.

[cit39] Fahim A. M., Dacrory S., Hashem A. H., Kamel S. (2024). Antimicrobial, anticancer activities, molecular docking, and DFT/B3LYP/LANL2DZ analysis of heterocyclic cellulose derivative and their Cu-complexes. Int. J. Biol. Macromol..

[cit40] Espinosa E., Alkorta I., Elguero J., Molins E. (2002). J. Chem. Phys..

[cit41] Nkungli N. K., Ghogomu J. N. (2017). Theoretical analysis of the binding of iron (III) protoporphyrin IX to 4-methoxyacetophe- none thiosemicarbazone via DFT-D3, MEP, QTAIM, NCI, ELF, and LOL studies. J. Mol. Model..

[cit42] Wojciechowska A., Szuster-Ciesielska A., Sztandera M. (2020). *et al.*). L-argininato copper(II) complexes in solution exert significant selective anticancer and antimicrobial activities. Appl. Organomet. Chem..

[cit43] (a) CCDC 2523709: Experimental Crystal Structure Determination, 2026, 10.5517/ccdc.csd.cc2qq3zk

