# Supplementary material for: Acetatobis(l-arginine)copper(ii) acetate trihydrate: synthesis from l-argininium acetate, structural-electronic insights and in vitro antimicrobial activity
Source: RSC Adv. 2026 Aug 27;16(42):50793–805. doi: 10.1039/d6ra03860c (PMC13519980; doi:10.1039/d6ra03860c)
Supplement: Supplementary file 1 [file RA-016-D6RA03860C-s001.pdf]

Supplementary Information (SI) for RSC Advances.
This journal is © The Royal Society of Chemistry 2026

Electronic supplementary information file

for the manuscript entitled:

Acetato bis(L-arginine)copper(II) acetate trihydrate: Synthesis from L-argininium acetate, structural-electronic insights and *in vitro* antimicrobial activity

Amani Direm[1,2*], Salima Samai[1,2], Hamza Athmani[1,2], Mohammed S. M. Abdelbaky[3,4], Cemal Parlak[5], Olufunso Abosede[6], Santiago Garcia-Granda[4] & Ponnadurai Ramasami[7,8*]

[1] Laboratory of Structures, Properties and Interatomic Interactions LASPI²A, Faculty of Sciences and Technology, Abbes Laghrour University, Khenchela 40000, Algeria.

[2] Department of Matter Sciences, Faculty of Sciences and Technology, Abbes Laghrour University, Khenchela 40000, Algeria.

[3] Departamento de Química Física, Facultad de Ciencias Químicas, Universidad de Salamanca, E-37008 Salamanca, Spain.

[4] Departamento de Química Física y Analítica, Universidad de Oviedo-CINN, 33006 Oviedo, Spain.

[5] Department of Physics, Science Faculty, Ege University, Izmir 35040, Turkey.

[6] Department of Chemistry, Federal University Otuoke, P.M.B 126, Yenagoa, Bayelsa State, Nigeria.

[7] Computational Chemistry Group, Department of Chemistry, Faculty of Science, University of Mauritius, Réduit 80837, Mauritius.

[8] Centre for Natural Product Research, Department of Chemical Sciences, University of Johannesburg, Doornfontein 2028, South Africa.

* Corresponding Author	: Amani Direm (ORCID : 0000-0002-6347-9173)
E-mail	: direm.amani@univ-khenchela.dz, amani_direm@yahoo.fr
Tel.	: +213.772.33.02.87
	: Ponnadurai Ramasami
E-mail	: p.ramasami@uom.ac.mu
Tel.	: +230 4037507

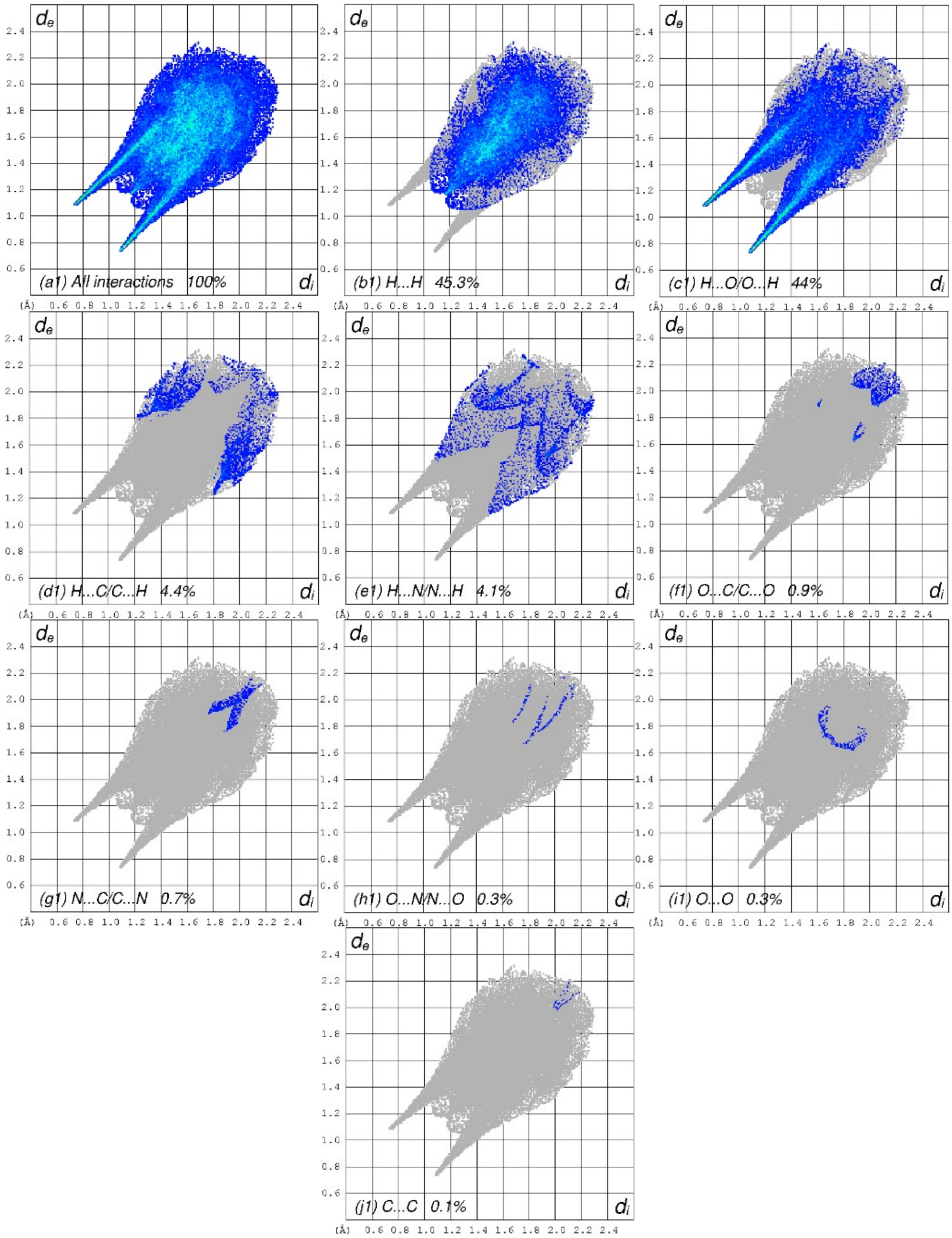

Figure S1. 2D-**FP** plots of the overall interactions in decomposed contacts' contribution in the structure of (**I**).

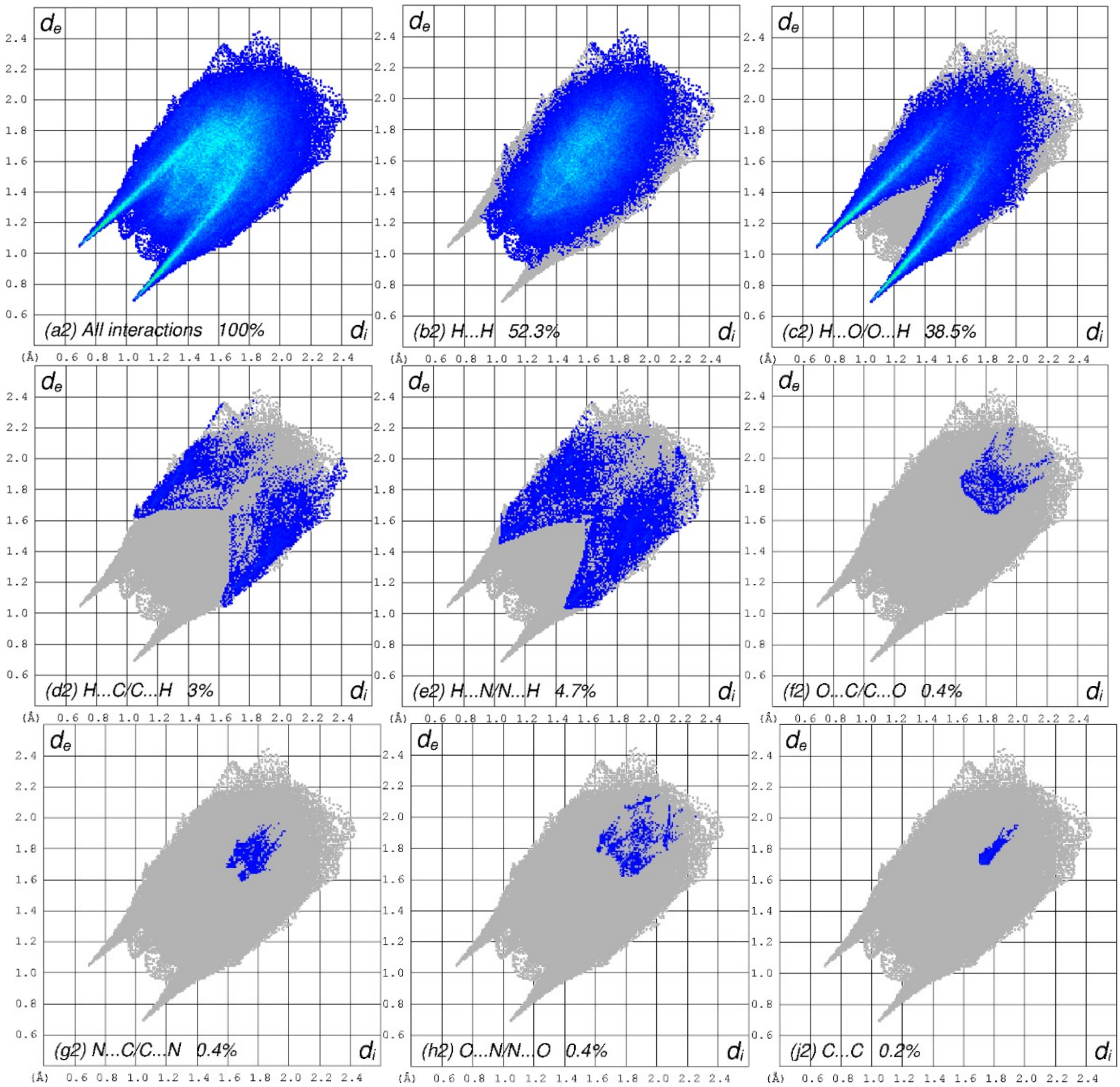

Figure S2. 2D-**FP** plots of the overall interactions in decomposed contacts' contribution in the structure of (**II**).

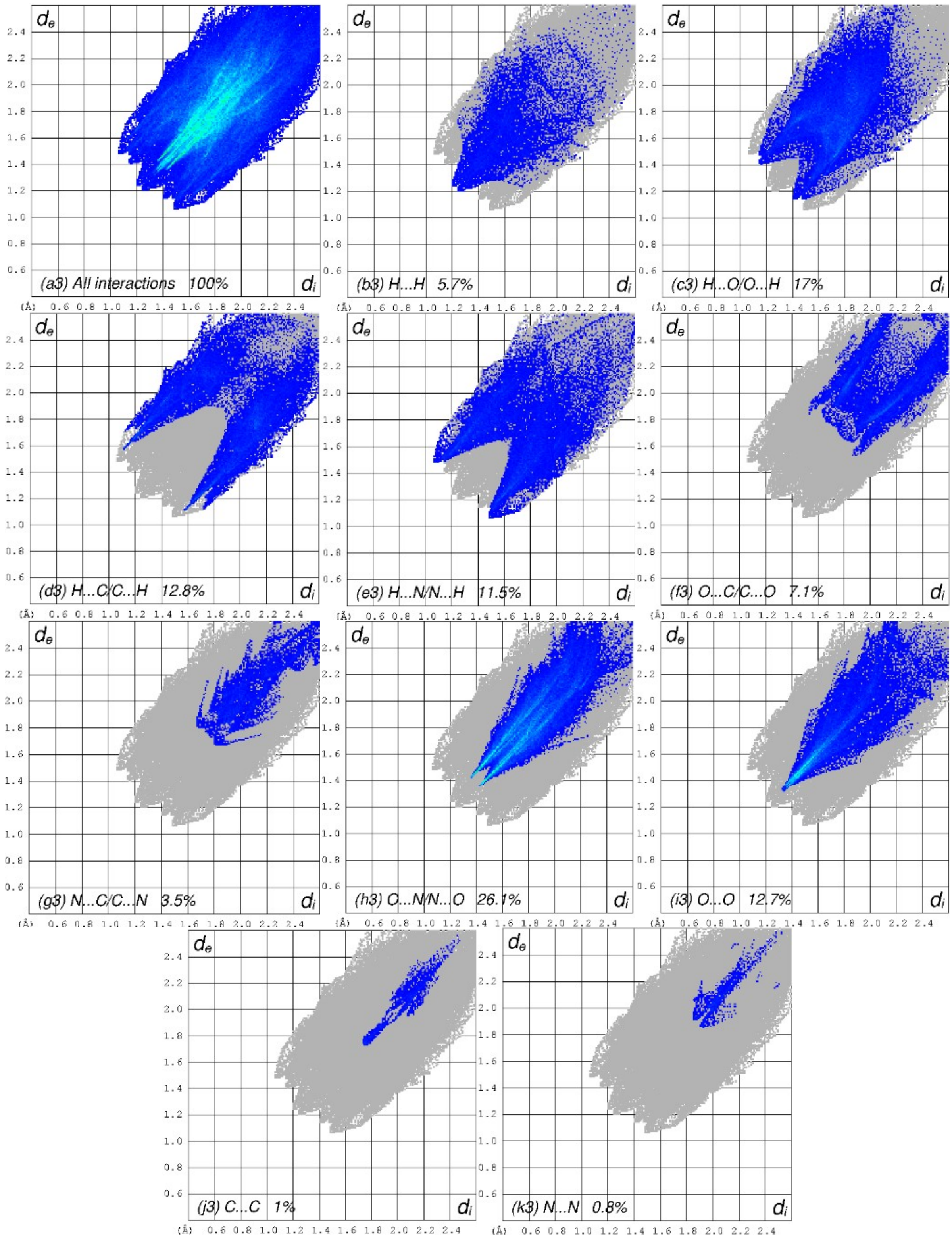

Figure S3. 2D-**FP** plots of the overall interactions in decomposed contacts' contribution in the structure of **DUYCAB**.
